# Oxidative stress leads to the formation of esterified *erythro-* and *threo-*dihydroxy-fatty acids in HepG2 cells

**DOI:** 10.1016/j.redox.2025.103589

**Published:** 2025-03-10

**Authors:** Lilli Scholz, Luca M. Wende, Michel A. Chromik, Nadja Kampschulte, Nils Helge Schebb

**Affiliations:** Chair of Food Chemistry, Faculty of Mathematics and Natural Sciences, University of Wuppertal, Gaussstrasse 20, 42119, Wuppertal, Germany

**Keywords:** Liquid chromatography, Tandem mass spectrometry, Oxylipins, Isoprostane, Eicosanoids

## Abstract

Oxidative stress plays a central role in pathophysiology. To assess oxidative stress, sensitive methods are required to monitor the cellular damage caused by reactive oxygen species. Phospholipid-bound isoprostanes, such as 5-iPF_2α_-VI determined by liquid chromatography-tandem mass spectrometry (LC-MS/MS) are currently the best markers for oxidative stress. Here, we describe *erythro-* and *threo-*dihydroxy-polyunsaturated fatty acids (PUFA), the hydrolysis products of *trans*- and *cis*-epoxy-PUFA, as new biomarkers of oxidative stress in cells. This is demonstrated in four oxidative stress models in HepG2 cells using radical-forming *tert-*butyl hydroperoxide, glutathione peroxidase 4 inhibiting RSL-3, the redox cycling agent paraquat or rotenone blocking the electron transport chain. LC-MS/MS analysis following the liberation of esterified oxylipins by saponification unveiled in all oxidative stress models a strong formation of *erythro-* and *threo-*dihydroxy-PUFA. The levels increased concentration-dependently and correlated to isoprostane 5-iPF_2α_-VI formation. Among the positional isomers derived from linoleic acid, arachidonic acid and docosahexaenoic acid, those bearing the dihydroxy-group closest to the carboxy terminus were predominantly formed. Thus, the highest concentrations were found of e*rythro*-5,6-DiHETrE and *erythro-*4,5-DiHDPE, which allowed a more sensitive detection of oxidative stress compared to 5-iPF_2α_-VI levels. The (*erythro-*) dihydroxy-PUFA are a new set of markers, which enable a more comprehensive analysis of oxidative stress, particularly when combined with simultaneous LC-MS/MS quantification of *trans-*epoxy-PUFA and isoprostanes.

## 1 Introduction

Oxidative stress is characterized by the imbalance between free radical reactive oxygen and nitrogen species (RONS) and the capacity of the cellular antioxidant defense. It plays a central role in the pathogenesis of many acute and chronic diseases such as neurodegenerative disorders, cancer as well as the aging process [1,2].

Oxidative stress is caused in cells by (i.) external stressors such as toxins or extracellularly formed free radicals, (ii.) enzymatic production of reactive oxygen species (ROS) or blockade of ROS detoxifying enzymes, (iii.) intracellular production of ROS by redox-cycling agents or (iv.) leakage of superoxide anions from the mitochondrial oxidative phosphorylation chain [3,4]. For the investigation of these pathways, different cell culture models have been established. To induce exogenous radical challenge, (i.) *tert-*butyl hydroperoxide (*t-*BuOOH) is directly added to the cell culture medium [5]. The inhibitor of the glutathione peroxidase 4 (GPX4) RSL-3 leads to an increase in hydroperoxy-polyunsaturated fatty acids (PUFA) driving membrane lipid peroxidation (ii.). GPX4 inhibition by RSL-3 together with an increase in iron further results in cell death so called ferroptosis [6,7]. For oxidative stress causing redox-cycling, (iii.) paraquat is commonly used as chemical probe. Here, the cycling of the paraquat dication to cation radical reaction consumes large amounts of NADPH and gives rise to superoxide anions. These can be converted to hydrogen peroxide and later hydroxyl radicals by the Fenton reaction [[8], [9], [10]]. An established method to investigate mitochondrial leakage of ROS (iv.) uses rotenone to stop the reverse electron flow from complex II to complex I of the electron transport chain in the mitochondrial membrane [11,12]. As a consequence, increased superoxide anions are produced and ATP as well as glutathione are depleted [11,13]. Of note, the rotenone model is used to investigate Parkinson's disease in rats developing disease-associated features such as systemic mitochondrial impairment and oxidative damage [12]. Due to this, the model is also used to mimic cell aging in the context of the “mitochondrial oxidative stress theory of aging” [2,14].

Key for the understanding and investigation of oxidative stress *in vivo* or in cell culture models are analytical methods detecting oxidative damage as sensitive and early as possible. During advanced oxidative stress, many biomolecules are oxidized, leading to modified proteins, DNA and lipids [15]. Among these targets of free radical reactions, PUFA are especially susceptible to (free radical-mediated) autoxidation [16]. The initial radical-mediated abstraction of an H-radical from bis-allylic positions of the PUFA-chain results in a mesomerically stabilized pentadienyl-like radical structure [17]. This reacts with molecular oxygen to peroxy-radicals, propagating the chain reaction, finally leading to a large number of stable and instable products [17,18].

Besides low-molecular weight lipid peroxidation end products such as 4-hydroxynonenal and malondialdehyde [18,19], arachidonic acid (ARA) gives rise to prostaglandin-like isoprostanes [20,21]. The correlation of oxidative stress and their formation is well established in various (animal) models [20,22]. Isoprostanes are formed *in situ* in phospholipids without a release of the fatty acid and independently from enzyme activity, such as cyclooxygenases [22]. Today, isoprostanes are established biomarkers for oxidative stress in different pathologies [21,22] and are linked to oxidative stress-related diseases, such as chronic obstructive pulmonary disease, inflammatory rheumatic diseases and cirrhosis [21,23]. However, also lifestyle habits like smoking and alcohol consumption can lead to increased isoprostane formation [21,23]. Based on this, these lipid peroxidation products are the best-established and most sensitive endpoints for monitoring oxidative stress [24,25].

However, in addition to isoprostanes, several other lipid peroxidation products are formed without a fission of the fatty acid backbone. Recently, we described that epoxy-PUFA are formed by ROS and may serve as oxidative stress markers [26]. In addition to *cis-*(*R,S-* and *S,R-*enantiomers)-epoxy-PUFA formed by cytochrome P450 monooxygenases, oxidative stress gives rise to both *cis-* and *trans-*(*R,R*- and *S,S*-enantiomers)-epoxy-PUFA [17,26].

In the present study, we show that also the hydrolysis products of *trans-* and *cis-*epoxy-PUFA, namely *erythro-* and *threo-*dihydroxy-PUFA are formed during autoxidation. Using HepG2 cells and four different treatments reflecting the different pathways of oxidative stress (see above), we demonstrate that particularly *erythro-*dihydroxy-PUFA are formed and allow a more sensitive detection of oxidative stress compared to established isoprostanes. Thus, we describe a new set of markers, consisting of epoxy-PUFA and dihydroxy-PUFA, which enable a more comprehensive analysis of oxidative stress in addition to the quantification of established isoprostanes.

## 2 Material and methods

### 2.1 Cell culture

HepG2 human liver carcinoma cells obtained from the German Collection of Microorganisms and Cell Cultures GmbH (DSMZ, Braunschweig, Germany) were grown in Eagle's Minimum Essential Medium supplemented with 10% fetal calf serum (superior standardized), 100 μM non-essential amino acids (l-alanine, l-asparagine, l-aspartic acid, l-glutamic acid, glycine, l-proline, l-serine), 2 mM l-glutamine, 100 U mL^−1^ penicillin and 100 μg mL^-1^ streptomycin in 60.1 cm^2^ dishes in a humidified incubator at 37 °C and 5% CO_2_.

For experiments, cells were seeded at densities of 0.25 × 10^6^ cells mL^−1^ 48 h before cell harvest. Cells were incubated with (i) 25–75 μM *t*-BuOOH in phosphate-buffered saline (PBS) for 4 h, (ii) 0.5–3 μM RSL-3 in DMSO for 4 h, (iii) 10–30 μM paraquat in PBS for 24 h and (iv) 0.05–1 μM rotenone in DMSO for 24 h. For vehicle control, cells were incubated with 0.1 % PBS resp. DMSO. Cells were harvested by gentle scraping and stored at −80 °C until analysis.

Cytotoxic effects of the test compounds were excluded by resazurin (alamar blue) assay [27], neutral red assay [28] and lactate dehydrogenase assay at the used concentrations. The tested compounds showed no cytotoxic effects (≥60 % of control) in the here used concentrations of *t-*BuOOH (25–75 μM, 4 h), RSL-3 (0.5–1 μM, 4 h), paraquat (10–30 μM, 4 h) and rotenone (0.05–1 μM, 4 h).

### 2.2 Quantification of free and total oxylipin levels by LC-MS/MS

Analysis of free (non-esterified) and total, i.e. sum of free and esterified oxylipins in cells was carried out as described [25,29,30]. In brief, HepG2 cell pellets (comprising approx. 5 × 10^6^ cells) were resuspended in 250 μL water/methanol (50/50, *v,v*) containing 10 μL antioxidant solution (0.2 mg/mL BHT, 100 μM indomethacin, 100 μM soluble epoxide hydrolase inhibitor *trans*-4-[4-(3-adamantan-1-yl-ureido)-cyclohexyloxy]-benzoic acid (t-AUCB) in methanol) and sonicated using an ultrasonic tip [30]. For free and total oxylipin analysis, deuterium-labeled oxylipins serving as internal standards (IS, all IS purchased from Cayman Chemicals, local distributor: biomol, Hamburg, Germany) were added to 100 μL of the cell homogenate before proteins were precipitated at −80 °C for at least 30 min using methanol or isopropanol, respectively. After centrifugation (20 000×*g*, 10 min, 4 °C), the supernatant served as sample for oxylipin analysis. Following alkaline hydrolysis (100 μL 0.6 M KOH (25/75, H_2_O/methanol, v/v) for 30 min at 60 °C) and solid-phase extraction on mixed-mode (non-polar and anion exchange) OasisMAX cartridges (3 mL, 60 mg sorbent per cartridge, particle size 30 μm, Waters, Eschborn, Germany) as described [31,32], total oxylipins were analyzed by targeted LC-MS/MS (QTRAP 5500, Sciex, Darmstadt, Germany) [30]. For the extraction of free oxylipins, samples were directly added onto the OasisMAX cartridges without alkaline hydrolysis [33] and analyzed the same way. Protein content was determined by bicinchoninic acid assay from the cell homogenate [34,35] and oxylipin concentrations were calculated as pmol/mg protein.

For the mass spectrometry characterization of *erythro-* and *threo-*DiHOME (Fig. S3), EDTA plasma from healthy human subjects was used (Supplementary Material). Plasma samples were prepared for analysis as described for homogenized cells.

Quantification of oxylipin concentrations was performed using external calibration standards to corresponding IS area ratios (linear fitting with 1/x^2^ weighting, Table S1) [25]. *Erythro-*dihydroxy-PUFA were quantified using the calibration of the corresponding *threo-*dihydroxy-PUFA [30]. *Erythro-* and *threo-*4,5-DiHDPE were semi-quantified relatively to 7,8-DiHDPE calibration.

## 3 Results and discussion

Oxidative stress is believed to play an important role in the etiology of numerous diseases [36]. For the assessment of oxidative stress several assays have been developed, however it is key to assess the onset of oxidative stress using sensitive analytical methods. So far, lipid peroxidation products resulting from membrane autoxidation such as isoprostanes are the most sensitive markers [24,37]. Here, *erythro*- and *threo*-dihydroxy-PUFA are introduced as a new class of lipid peroxidation products serving as biomarkers for oxidative stress. These were characterized in comparison to isoprostanes using four different oxidation models in HepG2 cells.

### 3.1 Characterization of *erythro*-dihydroxy-PUFA signals

HepG2 cells were used to characterize new oxidation products of PUFA formed during oxidative stress. Applying an LC-MS-based strategy, which has been successfully employed to uncover new oxylipins in vegetable oil [[38], [39], [40]], signals at *m/z* plus 16 (+1 O) or plus 34 (+2 OH) of the [M-H^+^]^-^ ions of PUFA were analyzed using an LC-MS method optimized for the separation and detection of oxylipins [25,29,30].

**Fig. 1:**
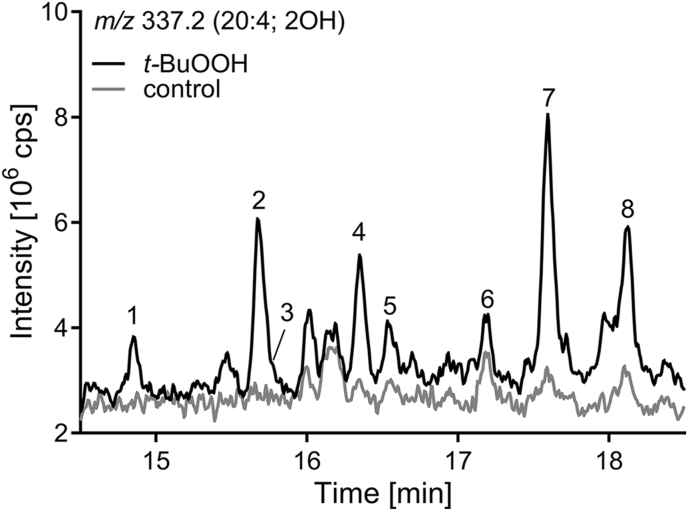
**Oxidized ARA in HepG2 cell extracts.** Cells were incubated with and without (control) *tert*-butyl hydroperoxide (*t*-BuOOH, 200 μM) for 4 h. Shown is the selected ion monitoring (SIM) LC-MS chromatogram of ARA + 2 OH at *m/z* 337.2. Eight peaks (1–8) were detected (black line) which were not found in in the control incubations.

Following incubation of HepG2 cells with radical generating *t*-BuOOH, we observed several new peaks at *m/z* 337.2 (20:4; 2OH, peaks 1–8, Fig. 1), *m/z* 313.2 (18:2; 2OH, peaks 1–4, Fig. S1) and *m/z* 361.2 (22:6; 2OH, peaks 1–12, Fig. S1). These peaks were absent or clearly less abundant in control incubations. Retention times and *m/z* in ESI(−)-MS of peaks 3 and 4 (*m/z* 313.2), 3, 5, 6 and 8 (*m/z* 337.2) and 3, 6, 7, 8, 10 and 12 (*m/z* 361.2) correspond to those of synthetic standards of *threo-* (*R,R-*/*S,S-*) dihydroxy-LA, -ARA and -DHA respectively (Fig. 1, S1) [30]. The unknown peaks 1 and 2 (*m/z* 313.2, Fig. S1); 1, 2, 4 and 7 (*m/z* 337.2, Fig. 1) as well as 1, 2, 4, 5, 9 and 11 (*m/z* 361.2, Fig. S1) elute about 0.5–0.9 min earlier (Table S1).

**Fig. 2:**
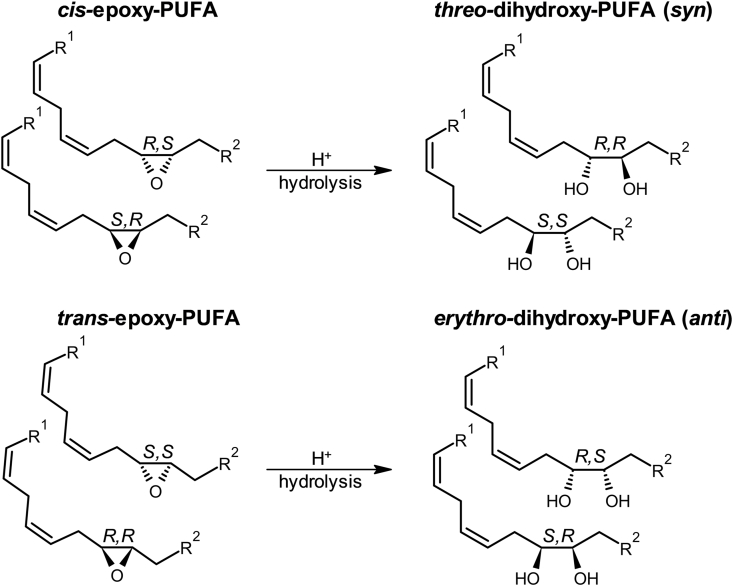
**Formation of *erythro-* and *threo-*dihydroxy-PUFA by hydrolysis of *trans-* and *cis*-epoxy-PUFA.** The two *cis-(R,S; S,R)-*epoxy-PUFA enantiomers lead to the formation of two *threo*-*(S,S; R,R)-*dihydroxy-PUFA enantiomers. Accordingly, the hydrolysis of *trans-(S,S; R,R)-*epoxy-PUFA leads to the formation of *erythro-(R,S; S,R)-*dihydroxy-PUFA.

In order to characterize the unknown peaks, we investigated the hydrolysis of epoxy-PUFA. As expected, acid-catalyzed hydrolysis of LA-derived *cis*-9(10)-EpOME led to the formation of a product with the same retention time and MS/MS-fragmentation behavior as the *threo-*9,10-DiHOME (*m/z* 313.2, t_R,a_ 14.9 min, Fig. S2) standard. Hydrolysis of a mixture of *trans-* and *cis*-12(13)-EpOME gave rise to two 12,13-DiHOME isomers (*m/z* 313.2, t_R___,___b_ 13.5 min, t_R,c_ 14.4 min, Fig. S2). In addition to *threo-*12,13-DiHOME, resulting from the hydrolysis of *cis-*12(13)-EpOME, the diastereomer *erythro-*12,13-DiHOME is formed from *trans-*12(13)-EpOME (Fig. 2).

The retention time and fragmentation behavior of the unknown peaks found in HepG2 cells as well as in human plasma (Fig. S3) are consistent with the formed hydrolysis product *erythro-*12,13-DiHOME (Fig. S2), indicating that *erythro-*12,13-DiHOME is formed in *t-*BuOOH-treated cells. According to these findings and the stereochemistry of epoxy-PUFA hydrolysis [41], we conclude that both *erythro-* and *threo-*dihydroxy-LA, -ARA and -DHA occur in biological samples, and result from hydrolysis of *cis-* and *trans-*epoxy-PUFA (Fig. 2).

**Fig. 3:**
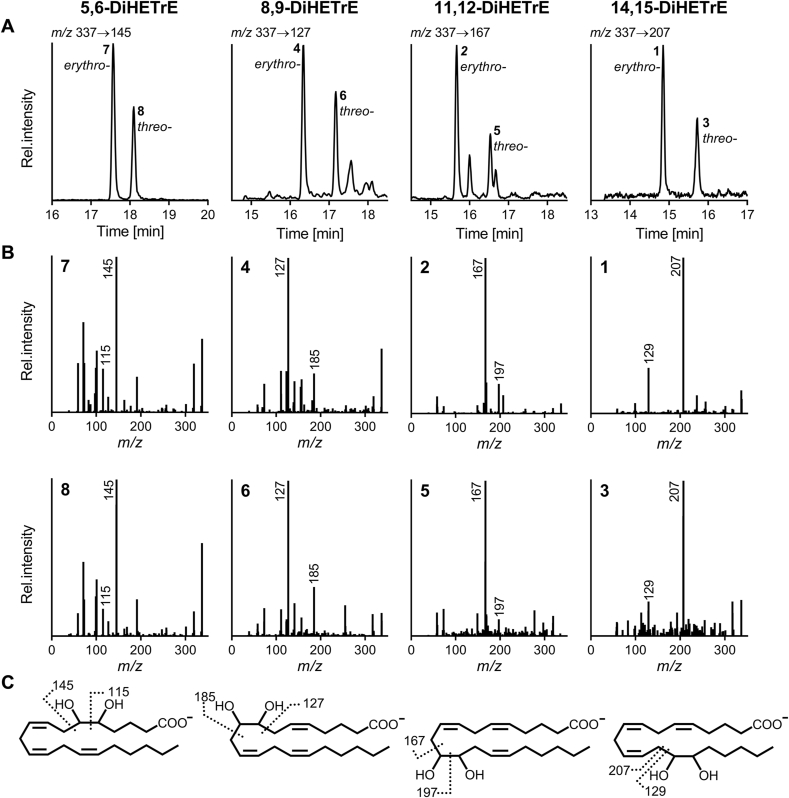
**LC-MS characterization of *erythro*- and *threo*-dihydroxy arachidonic acid positiona**l isomers. (**A**) Smoothed SRM chromatograms at the indicated transitions of the analysis of *tert-*butyl hydroperoxide (200 μM, 4 h) treated HepG2 cells. (**B**) Product ion spectra of the *erythro*- and *threo*-dihydroxy arachidonic acid (DiHETrE) eluting in peak 1–8 (Fig. 1). (**C**) Structures of the compounds and suggested sites of fragmentation by α-cleavage adjacent to the hydroxy groups are shown.

Further characterization of the peaks 1 and 2 (*m/z* 313.2, Fig. S1); 1, 2, 4 and 7 (*m/z* 337.2, Fig. 1) as well as 1, 2, 4, 5, 9 and 11 (*m/z* 361.2, Fig. S1) by MS/MS experiments revealed identical fragmentation behavior containing specific fragment ions derived from α-cleavage at the hydroxy-group(s) (Fig. 3, Figs. S3 and S4). This supports the characterization as *erythro-*dihydroxy-PUFA. All positional isomers of *erythro-* and *threo-*DiHOME (Fig. S3), -DiHETrE (Fig. 3) and -DiHDPE (Fig. S4) were found. Using an established reversed-phase chromatography method [25,29,30], a consistent elution pattern of the *erythro*/*threo*-stereoisomers was found (Fig. 1, Fig. 3). Elution of the *erythro-*dihydroxy-PUFA occurred 0.5–0.9 min earlier compared to the corresponding *threo-*dihydroxy PUFA isomers (Fig. 3, Fig. S3 and Fig. S4, Table S1). A comparable elution pattern has already been reported for *erythro-* and *threo-*dihydroxy-PUFA with 18 carbon atoms (octadecanoids) occurring in vegetable oils [38,40,42]. Here, we describe for the first time the occurrence of *erythro-*dihydroxy-PUFA in mammalian cells, in addition to the well-characterized *threo-*isomers.

**Fig. 4:**
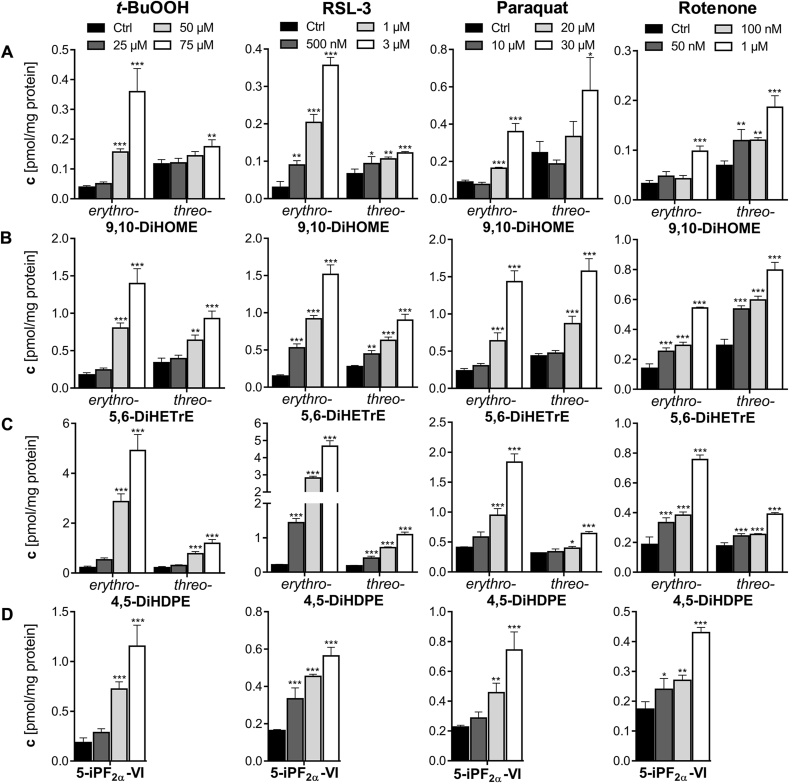
**Dose-dependent increase of *erythro-* and *threo-*dihydroxy fatty acids in four models of oxidative stress in HepG2 cells.** Cells were incubated with *tert-*butyl hydroperoxide (0–75 μM, 4 h, PBS as vehicle control), RSL-3 (0–3 μM, 4h, 0.1 % DMSO as vehicle control), paraquat (0–30 μM, 24 h, PBS as vehicle control) and rotenone (0–1 μM, 24 h, 0.1 % DMSO as vehicle control). Formation of *erythro-* and *threo-*dihydroxy-PUFA (**A**) 9,10-DiHOME, (**B**) 5,6-DiHETrE and (**C**) 4,5-DiHDPE in comparison to the formation of (**D**) the isoprostane 5-iPF_2α_-VI as established autoxidation marker. Total oxylipin concentrations in the cells were determined by LC-MS/MS (mean ± SD, n = 3). Significant increases compared to control incubations are indicated (∗p < 0.05; ∗∗p < 0.01; ∗∗∗p < 0.001) and were determined using one-way-ANOVA following Fisher's Least Significant Differences test.

**Fig. 5:**
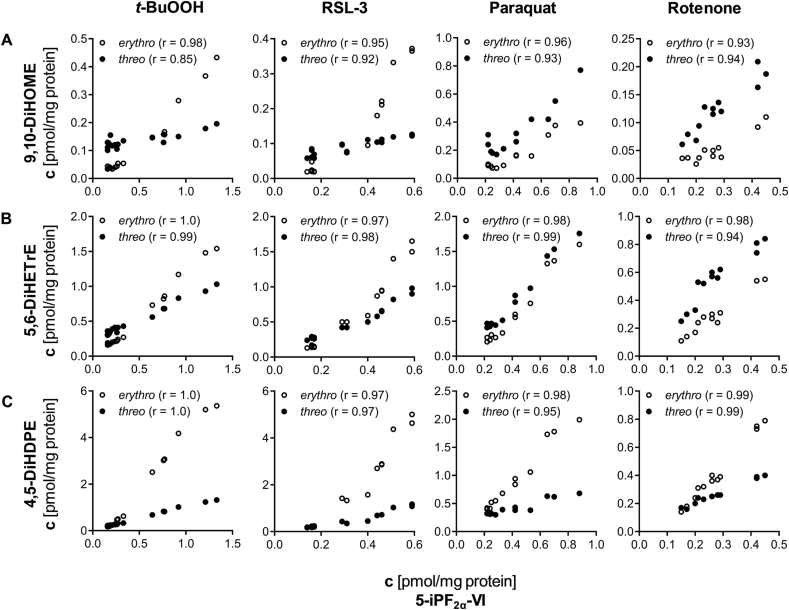
**Formation of *erythro-* and *threo-*dihydroxy-PUFA isomers correlates with isoprostane formation.***Erythro*- and *threo-* (**A**) 9,10-DiHOME, (**B**) 5,6-DiHETrE and (**C**) 4,5-DiHDPE are compared with 5-iPF_2α_-VI concentrations in different models of oxidative stress in HepG2 cells: *tert-*butyl hydroperoxide (0–75 μM, 4h, PBS as vehicle control), RSL-3 (0–3 μM, 4h, 0.1 % DMSO as vehicle control), paraquat (0–30 μM, 24 h, PBS as vehicle control) and rotenone (0–1 μM, 24 h, 0.1 % DMSO as vehicle control). Total oxylipin concentrations in the cells were determined by LC-MS/MS. Correlation of dihydroxy-PUFA to 5-iPF_2α_-VI concentrations was calculated using Pearson's r as indicated (p < 0.0001 for all correlations).

Comparing the formation of both diastereomers under oxidative stress conditions, we observed a stronger increase of *erythro-*dihydroxy-PUFA levels compared to *threo-*dihydroxy-PUFA diastereomers (Fig. 4, Fig. 5). This is in line with Rund et al. [26], describing a higher formation of *trans-*epoxy-PUFA during oxidative stress than the corresponding *cis-*epoxy-PUFA diastereomers. The observed oxidative stress-dependent formation of *erythro-*dihydroxy-PUFA thus seems to result from the hydrolysis of *trans-*epoxy-PUFA, while *threo-*dihydroxy-PUFA result from hydrolysis of *cis-*epoxy-PUFA (Fig. 2).

### 3.2 Formation of dihydroxy-PUFA in cells

At baseline and under oxidative stress conditions, dihydroxy-PUFA are present esterified to lipids in the cells. No or only minor (9,10- and 12,13-DiHOME <0.04 pmol/mg protein) concentrations of both *erythro-* and *threo-*dihydroxy-PUFA were detected as free (non-esterified) fatty acyls. This is similar to the occurrence of isoprostanes and epoxy-PUFA (Table S2) [22,26]. We only observed very small concentrations of non-esterified 9,10- and 12,13-DiHOME (2–28-fold lower than esterified) which is consistent with low concentrations of non-esterified 9(10)- and 12(13)-EpOME (5–20-fold lower than esterified) being the only epoxy-PUFA which could be detected as free fatty acyls (Table S2). In the used models of oxidative stress in HepG2 cells, isoprostanes, epoxy-PUFA and dihydroxy-PUFA were only found in relevant amounts esterified in the cells (Table S2, Table S3). Thus, the concentrations of total dihydroxy-PUFA (sum of esterified and non-esterified) directly reflect the concentrations of esterified ones and can be used for their quantification. Based on that, one can assume that the (esterified) isoprostanes, epoxy-PUFA and hydroxy-PUFA are formed directly from the esterified precursor PUFA and thus dihydroxy-PUFA are directly formed from esterified epoxy-PUFA without release and re-esterification.

This is supported by the finding that dihydroxy-PUFA formation in the used models is independent of the enzymatic hydrolysis by soluble epoxide hydrolase (sEH): In cells, non-esterified epoxy-PUFA are converted into dihydroxy-PUFA by enzymatic hydrolysis catalyzed by sEH [43]. sEH hydrolyzes both *trans-* and *cis-*epoxy-PUFA [44]. The used HepG2 cells express *EPHX2* and show sEH activity, which can be inhibited by the selective and potent sEH inhibitor TPPU (Fig. S1) [45,46]. However, the formation of total *erythro-* and *threo-*dihydroxy-PUFA in *t-*BuOOH-treated HepG2 cells was not affected by the inhibitor TPPU (Fig. S1). Since esterified epoxy-PUFA are no substrate for sEH [47], this clearly supports that the dihydroxy-PUFA are directly formed by non-enzymatic hydrolysis of esterified epoxy-PUFA.

In order to address in which lipid class, the autoxidation products are esterified, the lipids were fractionated in polar lipids and neutral lipids. Epoxy-PUFA, dihydroxy-PUFA as well as isoprostanes were found predominantly esterified to phospholipids in HepG2 cells (Fig. S6). Also in the case of oxidative stress, as exemplarily shown for HepG2 cells incubated with RSL-3, epoxy- and dihydroxy-PUFA were found in polar lipids, indicating an oxidation of the cellular membranes (Fig. S6).

Several oxylipins are potent lipid mediators. Cytochrome P450 monooxygenase-derived epoxy-PUFA show anti-inflammatory and vasodilatory effects [[48], [49], [50]]. However, these effects were only shown for free epoxy-PUFA. Thus, it is important to note that it is unclear if the epoxy-PUFA or their hydrolysis products formed by free radical-mediated oxidation of phospholipids are also acting as lipid mediators.

Membrane phospholipids are major targets of oxidative stress-induced lipid peroxidation [51]. In particular, PUFA esterified in phospholipids are prone to ROS-mediated membrane lipid peroxidation as the number of unsaturation increases [19,51]. Phospholipids bearing PUFA are mainly located in the inner leaflet of the plasma membrane and the mitochondrial membranes [52,53]. The PUFA ARA and DHA, which account for a high proportion of fatty acids in cells [54], are highly susceptible to free radical lipid peroxidation and therefore formation of epoxy-PUFA as well as isoprostanes have been frequently reported [22,26,52]. Here, we show that *erythro*- and *threo-*dihydroxy-PUFA are another class of oxylipins which are potential biomarkers for free radical membrane lipid peroxidation in cells, i.e. oxidative stress. The formation of *erythro-* and *threo-*dihydroxy derivatives occurs – compared to isoprostanes – in high concentrations (e.g. 0.92 ± 0.04 pmol/mg protein *erythro-*5,6-DiHETrE and 2.8 ± 0.1 pmol/mg protein *erythro-*4,5-DiHDPE vs. 0.46 ± 0.01 pmol/mg protein 5-iPF_2α_-VI and 0.070 ± 0.002 pmol/mg protein 8-iso-PGF_2α_ in RSL-3 treated HepG2 cells, Table S3). Moreover, several positional and stereo-isomers of LA, ARA and DHA can be detected (Fig. 4, Table S3). This not only enables a sensitive detection of oxidative stress, but also reduces the risk of an artificial detection of a single elevated lipid if a consistent and systematic formation of dihydroxy-PUFA from different precursors is found.

### 3.3 *Erythro*- and *threo*-dihydroxy-PUFA serve as oxidative stress markers

*Erythro-* and *threo-*dihydroxy-PUFA, *cis-* and *trans-*epoxy-PUFA as well as isoprostane formations were compared in four cell models of oxidative stress in HepG2 cells. All chemical probes for the investigation of oxidative stress were used in sub-cytotoxic concentrations (metabolic activity and lysosomal integrity >60 % of control, Fig. S8). Nevertheless, the elicited oxidative stress potently reduced cell proliferation at concentrations of 25 μM *t*-BuOOH (4 h), 10 μM paraquat (24 h) and 50 nM rotenone (24 h) (Fig. S7). The number of dead cells did not increase (Fig. S7), thus the reduced proliferation seems to be an effect of the oxidative stress. Treatment with RSL-3 did not reduce cell proliferation (Fig. S7). Although the investigated treatments led to different mechanisms of oxidative stress, they are all characterized by a concentration-dependent increase in *erythro-* and *threo-*dihydroxy-PUFA (Fig. 4, Table S3):

This is exemplarily shown for 9,10-DiHOME, 5,6-DiHETrE and 4,5-DiHDPE in Fig. 4. *Erythro-*dihydroxy-PUFA are formed in higher concentrations than *threo-*dihydroxy-PUFA (Fig. 4, Table S3). Among the regioisomers, the highest formation rate was observed for (*erythro-*) 5,6-DiHETrE and (*erythro-*) 4,5-DiHDPE (Fig. 4). Both, the *threo-* as well as the *erythro-*dihydroxy-ARA and -DHA showed a distinct pattern of the regioisomers with a stronger formation of those regioisomers in close proximity to the carboxy terminus esterified to phospholipids (Table S3).

Both *erythro-* and *threo-*dihydroxy-PUFA formation correlate well with 5-iPF_2α_-VI concentrations in all oxidative stress treatments (Fig. 5). This is consistent with our previous work, demonstrating that epoxy-PUFA, particularly *trans*-epoxy-PUFA, are markers of oxidative stress correlating with isoprostane formation [26]. In all four different oxidative stress models in HepG2 cells, the concentrations of *erythro-*dihydroxy-PUFA correlate with *trans-*epoxy-PUFA and *threo-*dihydroxy-PUFA correlate with *cis-*epoxy-PUFA (Fig. S9). The correlation of *erythro-* and *threo-*dihydroxy-PUFA and *trans-* and *cis-*epoxy-PUFA formation supports the formation of dihydroxy-PUFA from epoxy-PUFA in the membrane by non-enzymatic hydrolysis (Fig. 2, Fig. S9). Moreover, *erythro-*dihydroxy-PUFA increase stronger than *threo-*dihydroxy-PUFA during oxidative stress, and the latter show higher baseline levels. This is consistent with the higher formation of *trans*-epoxy-PUFA than *cis*-epoxy-PUFA, which also show higher baseline levels.

**Fig. 6:**
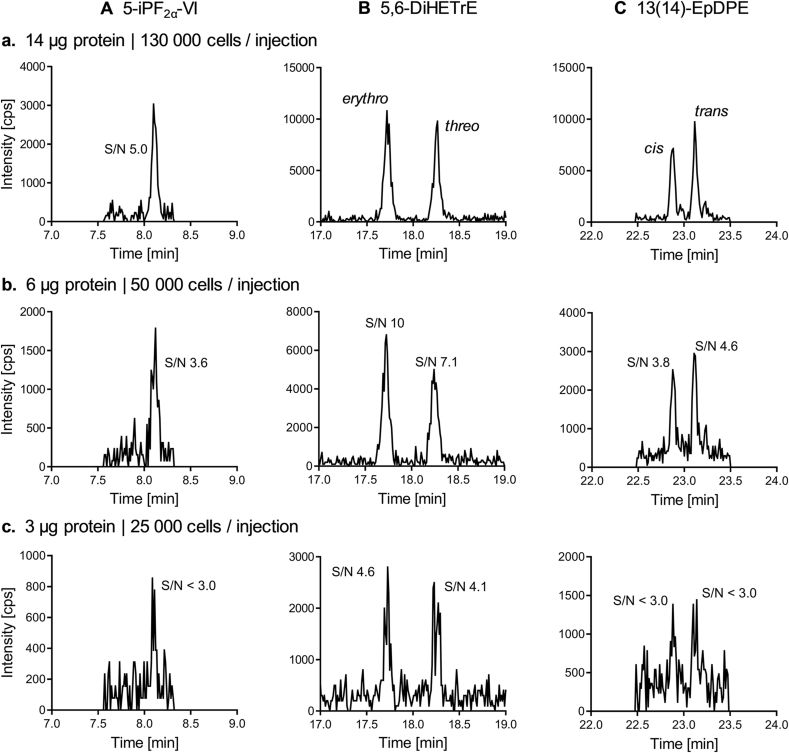
**Dihydroxy-PUFA and epoxy-PUFA are detected more sensitively than 5-iPF**_**2α**_**-VI requiring less sample material.** (**A**) 5-iPF_2α_-VI (0.3 pmol/mg protein), (**B**) 5,6-DiHETrE (0.5 pmol/mg protein *erythro*- and 0.4 pmol/mg protein *threo-*5,6-DiHETrE) and (**C**) 13(14)-EpDPE (0.3 pmol/mg protein *trans-* and 0.2 pmol/mg protein *cis-*13(14)-EpDPE) were analyzed in RSL-3 (500 nM, 4 h) challenged HepG2 cells by LC-MS/MS using samples corresponding to different cell numbers: Shown is the SRM signal of an injection of (**a**) 14 μg protein (approx. 130 000 cells), (**b**) 6 μg protein (approx. 50 000 cells) and (**c**) 3 μg protein (approx. 25 000 cells). The signal to noise ratio (S/N) of each peak is indicated if ≤ 10.

The lower limits of quantifications (LLOQ) are with 0.2 pg on column equal for the exemplarily shown analytes 5-iPF_2α_-VI, 13(14)-EpDPE and 5,6-DiHETrE in Fig. 6 [26,30,31]. However, oxidative stress induced by RSL-3 incubation (500 nM, 4 h) leads to higher concentrations of dihydroxy-PUFA compared to isoprostanes (Table S3) and thus the analysis of 5,6-DiHETrE is possible using a smaller amount of sample, i.e. cells (Fig. 6). Additionally, oxidative stress also results in stronger increases of dihydroxy-PUFA compared to isoprostanes and epoxy-PUFA (Fig. 4, Table S3). Epoxy-PUFA are observed at equal or higher concentrations, however their baseline concentrations are also higher (Table S3). The parallel quantification of *erythro-* and *threo-*dihydroxy-PUFA as well as *trans-* and *cis-*epoxy-PUFA allows a comprehensive and sensitive assessment of oxidative stress in cells. In all models, the increased levels of dihydroxy-PUFA were detected as soon as a reduced cell proliferation by oxidative stress was observed in HepG2 cells (Fig. 4, Fig. S7).

Determination of lipid peroxidation products, i.e. isoprostanes [37] by mass spectrometry detection is currently the most sensitive and reliable method, particularly when compared to immunoassays detecting isoprostanes and imaging tools using fluorescent dyes for the observation of ROS production indicating oxidative stress [24]. Here, we add dihydroxy-PUFA together with epoxy-PUFA, two classes of oxidized fatty acids, to the markers which depict oxidative stress. Parallel quantification of different regioisomers and diastereomers of dihydroxy- and epoxy-PUFA in addition to isoprostanes thus enables to gain a more sensitive, detailed and comprehensive picture of the ongoing oxidative processes in the cell.

## 4 Conclusion

In this study, *erythro-* and *threo-*dihydroxy-PUFA are characterized as new lipid peroxidation markers. Our data suggest that they are formed from PUFA in phospholipids by the hydrolysis of *trans-* and *cis-*epoxy-PUFA independently of enzymatic activities. Their presence in four models of oxidative stress using HepG2 cells challenged with (i.) *t*-BuOOH, (ii.) RSL-3, (iii.) paraquat and (iv.) rotenone, along with their strong correlation to isoprostane formation, underlines their potential as reliable indicators of oxidative damage. This is the first description of the occurrence of *erythro-*dihydroxy-PUFA in human cells. These *erythro-*dihydroxy-PUFA can be detected more sensitively than isoprostanes and thus offer new possibilities for the investigation of oxidative stress. Moreover, the analysis of dihydroxy-PUFA can be combined with that of isoprostanes and epoxy-PUFA offering a more comprehensive analysis of oxidative stress.

## CRediT authorship contribution statement

**Lilli Scholz:** Writing – original draft, Visualization, Investigation, Methodology, Conceptualization. **Luca M. Wende:** Writing – original draft, Methodology, Investigation. **Michel A. Chromik:** Writing – original draft, Methodology, Investigation. **Nadja Kampschulte:** Writing – original draft, Methodology. **Nils Helge Schebb:** Writing – original draft, Supervision, Conceptualization.

## Declaration of competing interest

The authors declare no financial or other competing intrests.[the funding listed here is not a financial intrest, and is acknowledged in the section above].

## Data Availability

The data is included in the manuscript/SI.

## Abbreviations

ARA: arachidonic acid
DHA: docosahexaenoic acid
DiHOME: dihydroxy-octadecenoic acid
DiHETrE: dihydroxy-eicosatrienoic acid
DiHDPE: dihydroxy-docosapentaenoic acid
EDTA: ethylenediaminetetraacetic acid
EpOME: epoxy-octadecenoic acid
EpETrE: epoxy-eicosatrienoic acid
EpDPE: epoxy-docosapentaenoic acid
IS: internal standard
LA: linoleic acid
LC-MS: liquid chromatography-mass spectrometry
LC-MS/MS: liquid chromatography-tandem mass spectrometry
LLOQ: lower limit of quantification
PBS: phosphate-buffered saline
ROS: reactive oxygen species
RONS: reactive oxygen and nitrogen species
RSL-3: ras selective lethal protein 3
sEH: soluble epoxide hydrolase
*t-*BuOOH: *tert*-butyl hydroperoxide
