# Supplementary material for: Oxidative stress leads to the formation of esterified erythro- and threo-dihydroxy-fatty acids in HepG2 cells
Source: Redox Biol. 2025 Mar 10;82:103589. doi: 10.1016/j.redox.2025.103589 (PMC11964660; doi:10.1016/j.redox.2025.103589)
Supplement: Multimedia component 1 [file mmc1.pdf]

Oxidative stress leads to the formation of *erythro-* and *threo*-dihydroxy-fatty acids in HepG2 cells

Lilli Scholz, Luca M. Wende, Michel A. Chromik, Nadja Kampschulte and Nils Helge Schebb*

Chair of Food Chemistry, Faculty of Mathematics and Natural Sciences, University of Wuppertal, Gaussstrasse 20, 42119 Wuppertal, Germany

*Corresponding author (Tel: +49 202-439-3457; E-mail: nils@schebb-web.de)

Supplementary material

Contents

1. Supplementary materials…………………………………………………………………… 4

2. Supplementary methods

Acid hydrolysis of epoxy-PUFA……………………………………………………………. 4

In vitro measurement of sEH activity in HepG2 cells……………………………….……. 4

Determination of sEH protein amount by targeted proteomics……………………….... 5

Tab. 1: Selected proteotypic peptides (PTPs) from in silico tryptic digest of EPHX2………………………………………………………………………………..… 5

Lipid fractionation method…………………………………………………...…………….. 6

Proliferation assay…………………………………………………………………………... 6

3. Supplementary figures:

Fig. S1: Selected ion monitoring LC-MS chromatograms of LA + 2 O (m/z 313.2) and DHA + 2 O (*m/z* 361.2) in HepG2 cell extracts……………………...………….……….. 7

Fig. S2: Acid hydrolysis of epoxy-PUFA to corresponding dihydroxy PUFA……….... 8

Fig. S3: LC-MS characterization of *erythro*- and *threo*-dihydroxy-octadecenoic acid positional isomers ……………………………………………………………………….... 9

Fig. S4: LC-MS characterization of *erythro*- and *threo*-dihydroxy-docosapentaenoic acid (DiHDPE) positional isomers ……………………………………………......…. 10

Fig. S5: Oxidative stress related formation of dihydroxy-PUFA is independent from soluble epoxide hydrolase (sEH) activity in HepG2 cells…………………….……….…. 11

Fig. S6: Dihydroxy- and epoxy-PUFA are esterified to phospholipids………………….. 12

Fig. S7: Oxidative stress limits cell proliferation…………………………………...….... 13

Fig. S8: Cell viability of HepG2 cells during oxidative stress treatments……………… 14

Fig. S9: Absolute concentrations of *trans*-epoxy-PUFA correlate with *erythro*-dihydroxy-PUFA concentrations and *cis*-epoxy-PUFA correlate with *threo*-dihydroxy-PUFA………………………………………………………………………………………. 15

4. Supplementary tables:

Tab. S1: Parameters of the LC-ESI(-)-MS/MS method for the quantification of *erythro*- and *threo*-dihydroxy-PUFA, cis- and trans-epoxy-PUFA and selected isoprostanes………………………………………………………….………………... 16

Tab. S2: Free oxylipins in models of oxidative stress……………………………………..….. 18

Tab. S3: Total oxylipins in models of oxidative stress……………………………..…..…... 20

1. Supplementary materials

2-Propanol, methanol (MeOH), acetonitrile and acetic acid (all MS grade) were purchased from Fisher Scientific (Schwerte, Germany). Ultrapure water was generated using the Barnstead Genpure Pro system from Thermo Fisher Scientific (Langenselbold, Germany). Eagle's Minimum Essential Medium was purchased from Fisher Scientific (Schwerte, Germany) and fetal calf serum (superior standardized) from Biochrom (Berlin, Germany). DMSO was purchased from Carl Roth (Karlsruhe, Germany). All other chemicals were purchased from Merck (Darmstadt, Germany). Oxylipin internal standards, the sEH inhibitor TPPU and *t*-AUCB as well as the COX inhibitor indomethacin were purchased from Cayman Chemical (local distributor: biomol, Hamburg, Germany). *Cis*-9(10)-EpOME and *cis*- and *trans*-12(13)-EpOME were purchased from Larodan (Stockholm, Sweden). Pooled human EDTA plasma from healthy individuals was generated as described [31], approved by the ethics committee of the University of Wuppertal and in accordance with the guidelines of the declaration of Helsinki. In brief, for plasma generation, blood was collected in EDTA-monovettes and immediately centrifuged for 10 min at 1200 x g and 4°C. The supernatant was carefully collected. Plasma samples from different subjects were pooled, gently mixed, aliquoted and frozen at -80°C immediately afterwards.

2. Supplementary methods

Acid hydrolysis of epoxy-PUFA

Standards of *cis*-9(10)-epoxy linoleic acid (9(10)-EpOME) and a mixture of *trans*-12(13)-EpOME and *cis*-12(13)-EpOME (0.5-1 µM) in 500 µL acetonitrile were hydrolyzed with 500 µL of 1 M aqueous H_2SO_4 for 24 h at room temperature. After neutralization with 500 µL of 1 M $NaHCO_3$ in water, oxylipins were extracted by liquid-liquid extraction using ethyl acetate and reconstituted in methanol following evaporation of the extraction solvent. Extracts were analyzed by LC-MS/MS for *erythro*- and *threo*-9,10-dihydroxy linoleic acid (9,10-DiHOME, *m/z* 313.2) and -12,13-DiHOME (*m/z* 313.2) as well as for their fragmentation behavior (MS^2, product ion scan).

In vitro measurement of sEH activity in HepG2 cells

For investigation of sEH activity in HepG2 cells, cell pellets were homogenized in PBS using an ultrasonic tip. Cell homogenates were pooled and aliquots of 200 µL were used for this assay. Protein content was determined by bicinchoninic acid assay. In this assay, a protein amount of 750 µg protein per 200 µL sample (3.8 mg/mL) was determined. Samples were stored on ice until incubation started. Pre-incubations with the sEH-inhibitor TPPU were carried

out for 5–10 minutes with 1 µL 200 µM TPPU in PBS yielding a final concentration of 1 µM TPPU. The activity assay was started by the addition of EpETrE substrates (11(12)- and 14(15)-EpETrE, 42 µM and 67 µM final concentration, respectively) and incubation was carried out at 30°C for 30 min. Incubations of HepG2 cell homogenate without the addition of EpETrE substrate with and without TPPU pre-incubation served for background measurements. The assay was stopped by the addition of ice-cold ethyl acetate. 17,18-DiHETE which is absent in HepG2 samples was added as internal standard. Dihydroxy metabolites were extracted twice by liquid-liquid extraction with ethyl acetate. Combined ethyl acetate phases were evaporated and reconstituted in methanol. 11,12- (m/z 337.2 → 167.1) and 14,15-DiHETrE (m/z 337.2 → 207.1) metabolites were analyzed by targeted LC-MS/MS and evaluated relatively to 17,18-DiHETE (internal standard, m/z 335.2 → 247.2).

Determination of sEH protein amount by targeted proteomics

Sample preparation for the quantification of sEH enzyme concentrations in HepG2 cells was performed according to Hartung *et al.* [46]. In brief, an aliquot of sonicated and lysed HepG2 cells containing 500 µg protein (protein content determined using bicinchoninic acid assay) was used for sample preparation consisting of acetone precipitation, reduction of disulfide bridges using dithiothreitol, alkylation of the free sulfhydryl groups using iodoacetamide and protein digestion using trypsin. Following addition of heavy labeled internal standards (JPT Peptides, Berlin, Germany) samples were subjected to solid-phase extraction (Strata-X 33 µm Polymeric Reversed Phase 100 mg per 3 mL, Phenomenex LTD, Aschaffenburg, Germany) and analyzed by LC-MS/MS.

Selection of proteotypic peptides for sEH quantification (Tab. 1) was carried out according to Hartung *et al.* [46].

Table 1: Selected proteotypic peptides (PTPs) from *in silico* tryptic digest of *EPHX2*. Shown are experimentally selected transitions of three PTPs (unlabeled and heavy labeled) for the quantification of the protein amount of sEH (*EPHX2*) including mass transitions, retention times and transition's intensity ratios determined by LC-MS/MS.

			unlabeled		*heavy labeled*			
**gene/ protein (Uniprot No.)**	**peptide**	**transitions**	**Q1 *m/z***	**Q3 *m/z***	**Q1 *m/z***	**Q3 *m/z***	**RT [min]**	**rel. ratio to quantifier [%]**
EPHX2 / soluble epoxide hydrolase (sEH) (P34913)	TEEALALPR	$M^{2+} \rightarrow y^{7+}$	500.3	769.5	505.3	779.5	12.4 ± 0.2	100
		$M^{2+} \rightarrow y^{6+}$		640.4		650.4		52
		$M^{2+} \rightarrow y^{5+}$		569.4		579.4		57
	VCEAGGLFVNS PEEPSLSR	$M^{2+} \rightarrow y^{5+}$	1024.5	559.3	1029.5	569.3	16.9 ± 0.2	100
		$M^{2+} \rightarrow y^{9+}$		1001.5		1011.5		47
	WLDSDAR	$M^{2+} \rightarrow y^{5+}$	431.7	563.2	436.7	573.3	9.9 ± 0.2	100
		$M^{2+} \rightarrow y^{2+}$		246.2		256.2		62
		$M^{2+} \rightarrow b^{2+}$		300.2		300.2		30

Lipid fractionation method

For the differentiation of oxylipins bound in neutral lipids and in phospholipids, the phospholipids were separated from the neutral lipids using an aminopropyl solid phase extraction strategy, and the oxylipins were determined in both fractions following saponification. Solid-phase extraction (SPE) for lipid class fractionation was performed using CHROMABOND NH_2 cartridges (45 µm, 3 mL/500 mg; Machery-Nagel, Düren, Germany). Cartridges were preconditioned with 6 mL of H_2O/acetonitrile (5/95, *v/v*). Then 1000 µL of acetonitrile and the sample were loaded onto the column.

Neutral lipids were eluted with H_2O/acetonitrile (5/95, *v/v*). Phospholipids were collected using H_2O/acetonitrile (11/89, *v/v*) and methanol containing 0.1% acetic acid.

Elution of the lipid fractions was conducted under a slight vacuum (~900 mbar). After collecting, 8 µL of 30% glycerol in methanol was added to each fraction, and solvents were evaporated under vacuum. The residues were reconstituted in pure isopropanol and then saponification, extraction and analysis were carried out according to the protocol for targeted quantification of total oxylipins by LC-MS/MS.

Proliferation assay

Effects on cell proliferation were investigated by counting the cells after 48 h. HepG2 cells were seeded in 6-well plates (250 000 cells/mL, 375 000 cells/well). After 24 h cells were treated with (i) *tert*-butyl hydroperoxide and (ii) RSL-3. After 4 h incubation, the medium was renewed and the cells were incubated for another 20 h before counting the cells. 24 h after seeding, cells were incubated with (iii) paraquat and (iv) rotenone for another 24 h before counting the cells using a Neubauer chamber (Fig. S6).

3. Supplementary figures

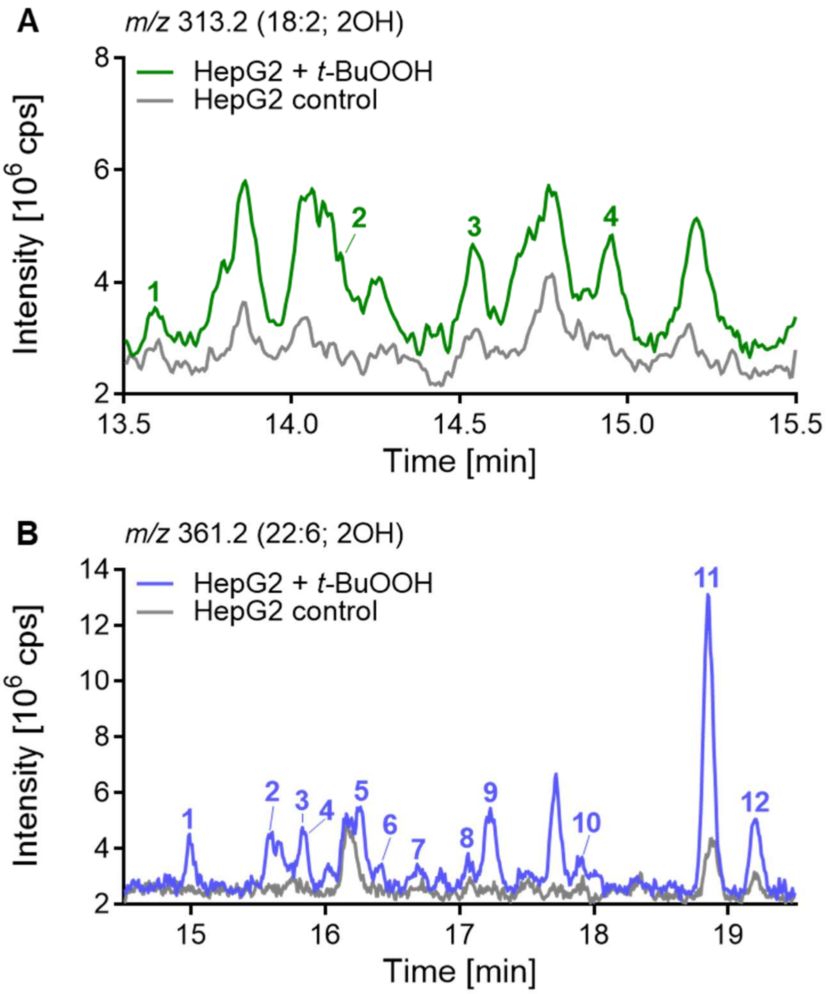

Fig. S1: Selected ion monitoring (SIM) LC-MS chromatograms of LA + 2 OH (*m/z* 313.2) and DHA + 2 OH (*m/z* 361.2) in HepG2 cell extracts. Cells were incubated with *tert*-butyl hydroperoxide (*t*-BuOOH, 200 µM) for 4 hours. 4 peaks (*m/z* 313.2, green line) and 12 peaks (*m/z 361.2*, blue line) were detected which were not found in the control incubation (grey line).

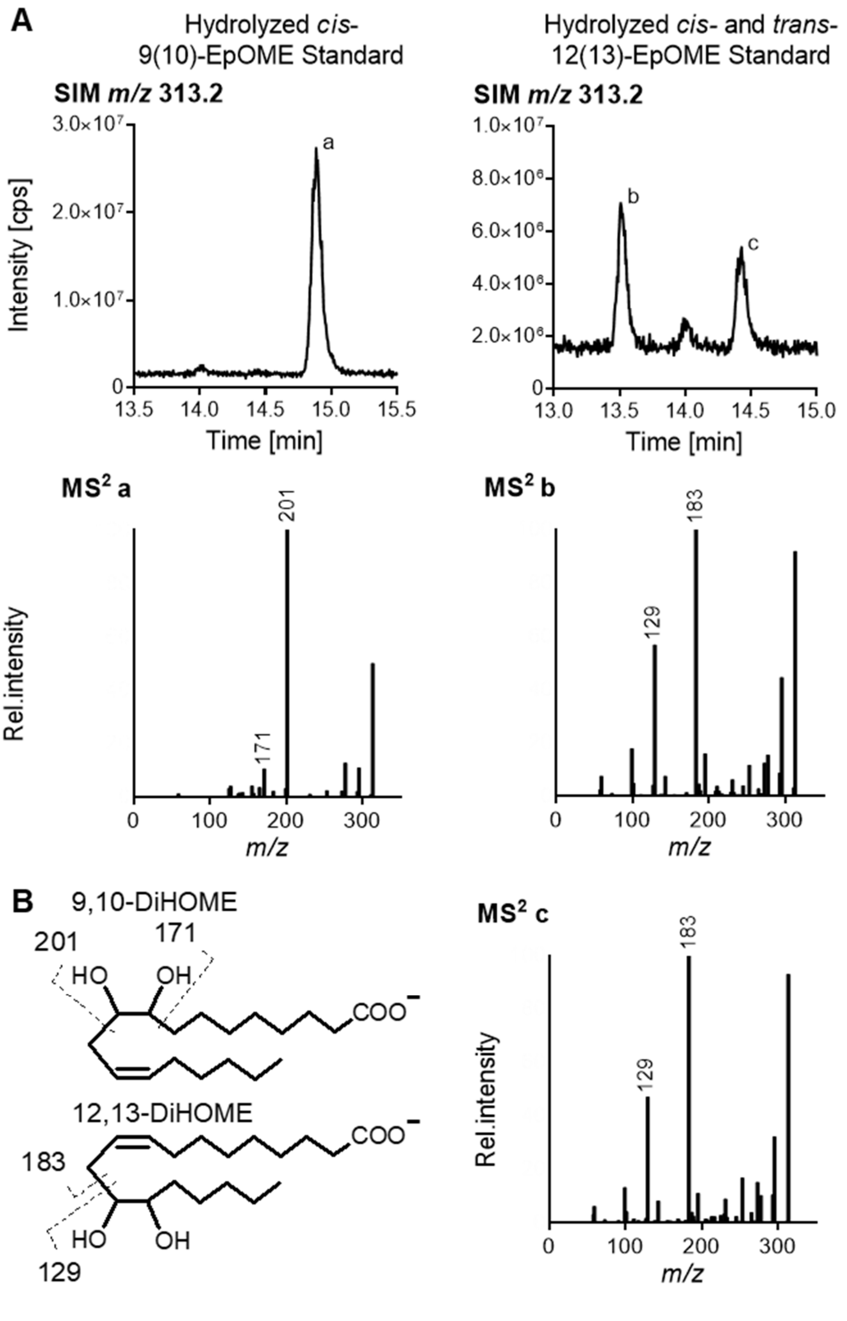

Fig. S2: Acid hydrolysis of epoxy PUFA to corresponding dihydroxy PUFA. Hydrolysis of *cis*-9(10)-epoxy octadecenoic acid (9(10)-EpOME) yields *threo*-9,10-dihydroxy octadecenoic acid (9,10-DiHOME) whereas hydrolysis of a mixture of *cis*- and *trans*-12(13)-epoxy octadecenoic acid (12(13)-EpOME) leads to both *threo*- and *erythro*-12,13-dihydroxy octadecenoic acid (12,13-DiHOME). Standards of *cis*-9(10)-EpOME and a mixture of *trans*-12(13)-EpOME and *cis*-12(13)-EpOME were hydrolyzed with H_2SO_4. After neutralization, oxylipins were extracted by liquid-liquid extraction and analyzed by LC-MS. (**A**) In selected ion monitoring of the corresponding dihydroxy products 9,10-DiHOME and 12,13-DiHOME (*m/z* 313.2) one signal after hydrolysis of *cis*-9(10)-EpOME and two signals after hydrolysis of the *cis*- and *trans*-12(13)-EpOME mixture are detected. Retention times and fragment spectra match with (a) *threo*-9,10-DiHOME, (b) *erythro*- and (c) *threo*-12,13-DiHOME. (**B**) Structures of DiHOME and suggested sites of fragmentation by α-cleavage adjacent to the hydroxy groups.

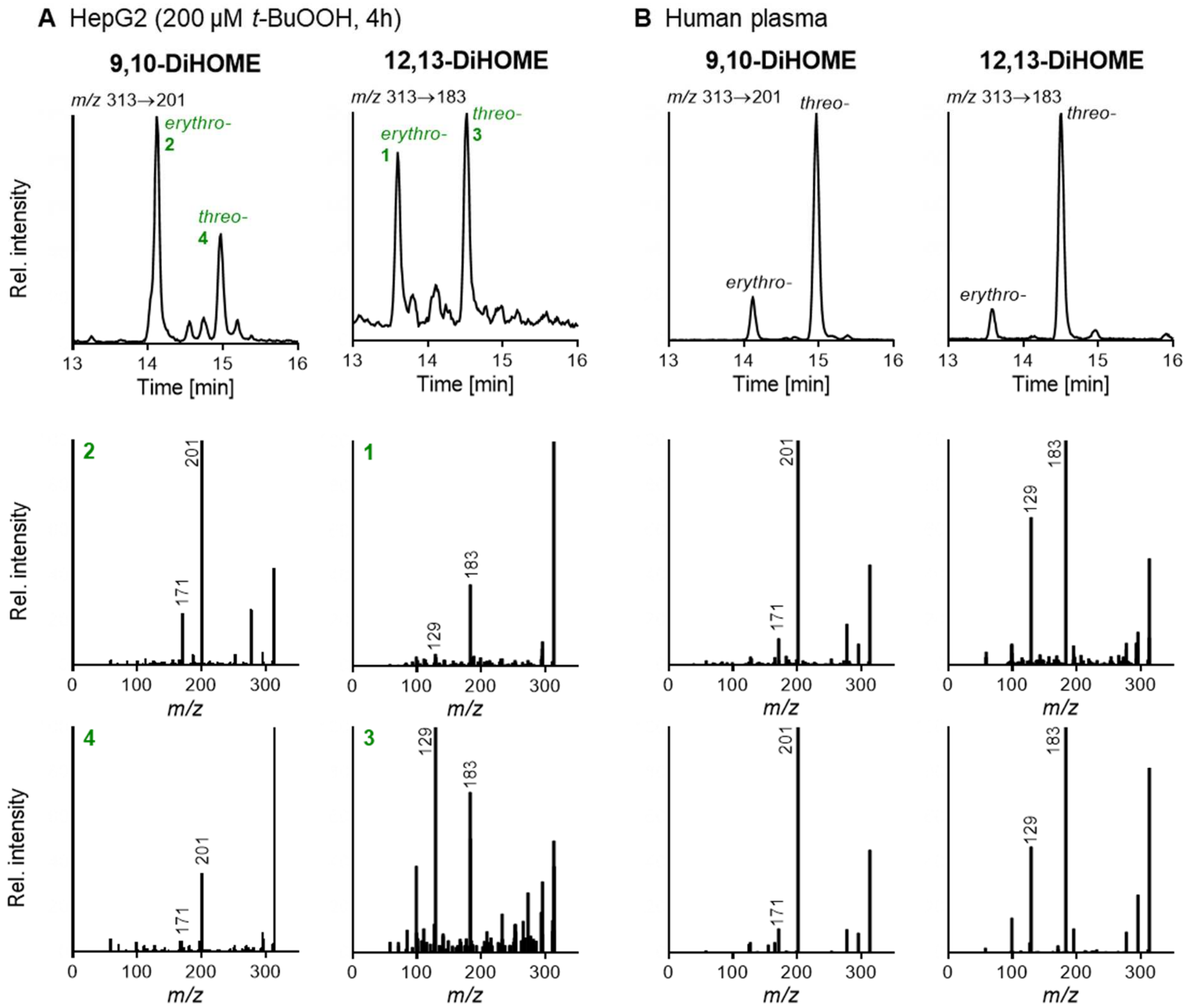

Fig. S3: LC-MS characterization of *erythro*- and *threo*-dihydroxy-octadecenoic acid positional isomers. Smoothed SRM chromatograms at the indicated transitions of the analysis of (**A**) *tert*-butyl hydroperoxide (200 µM, 4 h) treated HepG2 cells and (**B**) human plasma. Shown are corresponding product ion spectra of *erythro*- and *threo*-dihydroxy octadecenoic acid (DiHOME). Structures of the compounds and suggested sites of fragmentation by α-cleavage adjacent to the hydroxy groups are shown in Fig. S4. Fragments from α-cleavage are seen more clearly in the extracts of human EDTA plasma than of HepG2 cells due to higher abundance of the oxylipin. However, retention times of *erythro*- and *threo*-9,10-DiHOME and 12,13-DiHOME in HepG2 cells (peaks 1-4, Fig. S1, A) exactly match retention times in human plasma.

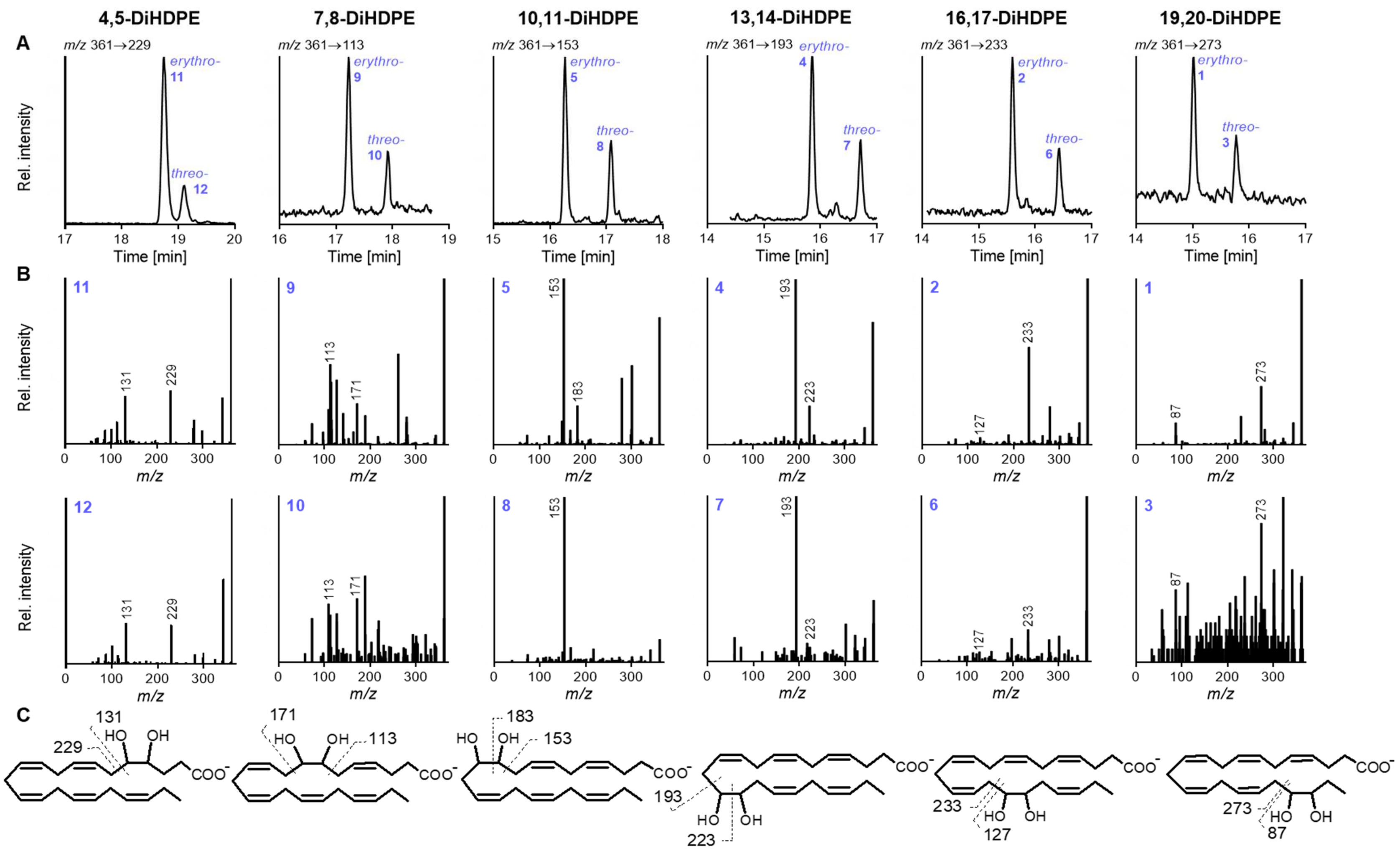

Fig. S4: LC-MS characterization of *erythro*- and *threo*-dihydroxy-docosapentaenoic acid (DiHDPE) positional isomers. (**A**) Smoothed SRM chromatograms at the indicated transitions of the analysis of *tert*-butyl hydroperoxide (200 µM, 4 h) treated HepG2 cells. (**B**) Corresponding product ion spectra of *erythro*- and *threo*-DiHDPE. (**C**) Structures of the compounds and suggested sites of fragmentation by α-cleavage adjacent to the hydroxy groups. Peak numbers (blue) correspond to Fig.S1, B.

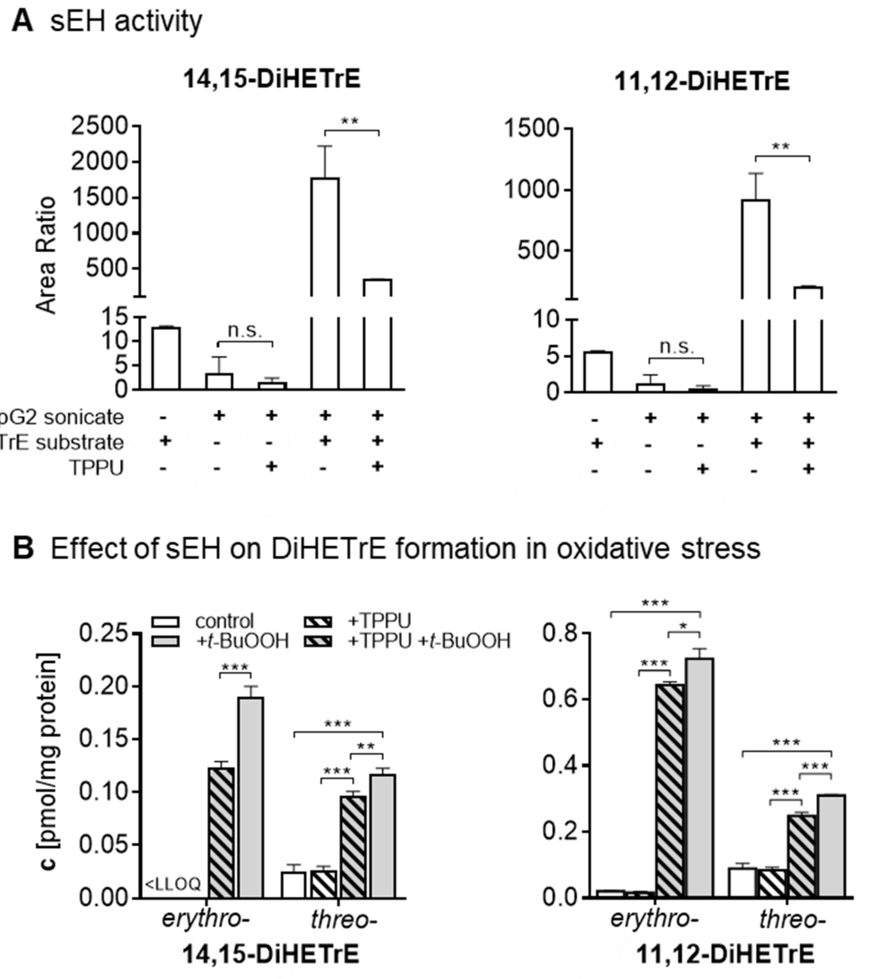

Fig. S5: Oxidative stress-related formation of dihydroxy-PUFA is independent of soluble epoxide hydrolase (sEH) activity in HepG2 cells. HepG2 cells contain sEH (5 ± 1 fmol/mg protein, mean ± SD, n = 3) as determined by targeted LC-MS/MS proteomics and we demonstrate that they show sEH activity (**A**) which can be inhibited by the sEH-inhibitor 1-trifluoromethoxyphenyl-3-(1-propionylpiperidin-4-yl) urea (TPPU): HepG2 cell pellets were homogenized in ice-cold PBS using an ultrasonic tip. For sEH inhibition, cell homogenates were pretreated with 1 µM TPPU for 10 min on ice. EpETrE substrates were added and samples were incubated at 30°C for 30 min. After incubation, samples were extracted twice with ethyl acetate after addition of 17,18-DiHETE as internal standard (IS). Extracts were analyzed for 14,15-DiHETrE and 11,12-DiHETrE by LC-MS/MS. Results are shown as area ratios relative to the IS (mean ± SD, n = 3). TPPU does not inhibit formation of total dihydroxy-PUFA during oxidative stress (**B**): In *tert*-butyl hydroperoxide treated HepG2 cells (50 µM, 3 h), oxidative stress-related formation of dihydroxy-PUFA and its dependence on sEH activity was investigated using the sEH-inhibitor TPPU. The cells were preincubated with TPPU (1 µM) 3h before *t*-BuOOH was added to the cell culture dish for another 3 h. Oxylipins were quantified by LC-MS/MS (mean ± SD, n = 3). Statistical significances were determined with a t-test (two-tailed, * $p < 0.05$, ** $p < 0.01$, *** $p < 0.001$).

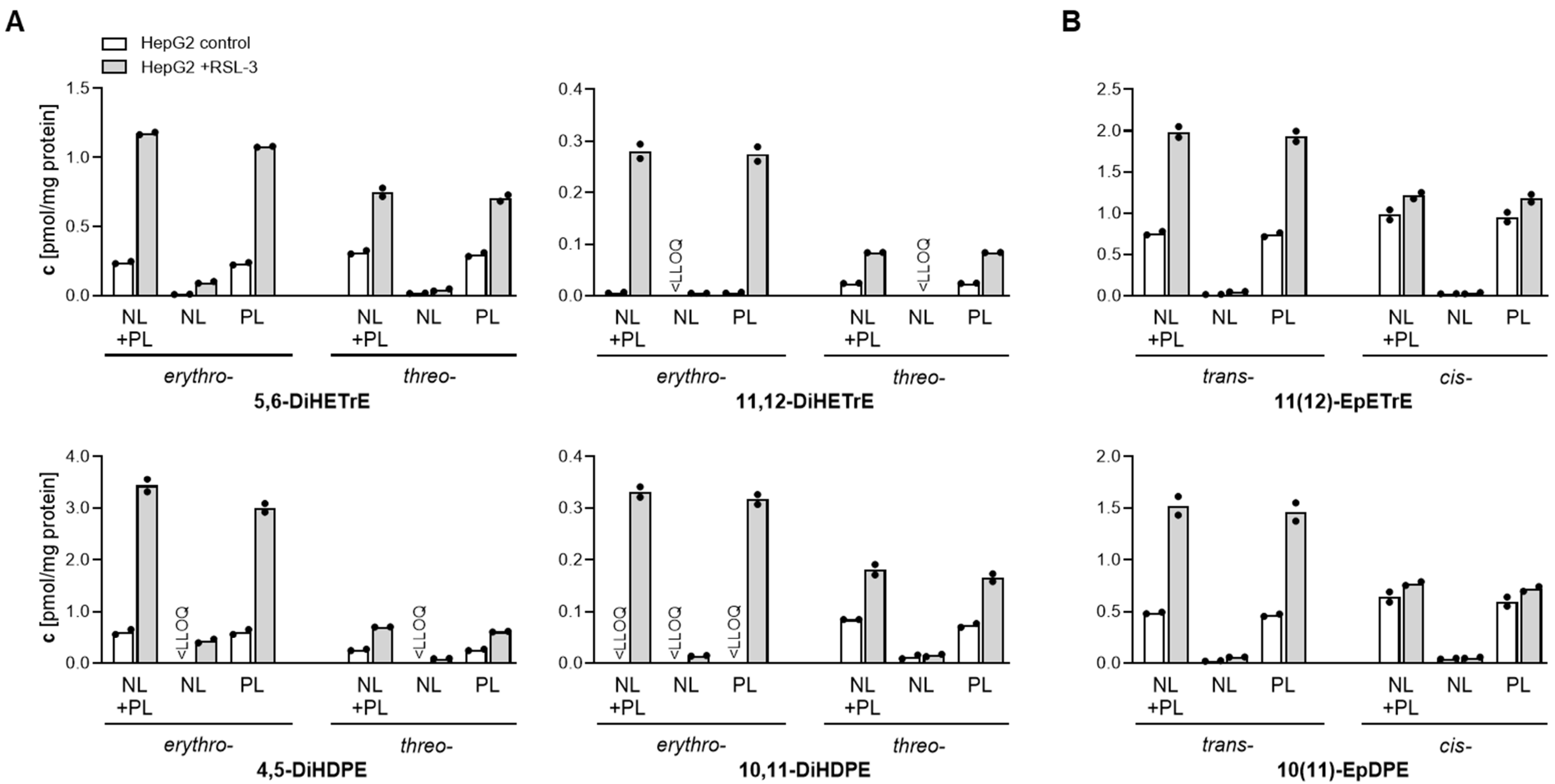

Fig. S6: Dihydroxy- and epoxy-PUFA are esterified to phospholipids. RSL-3 treated as well as untreated HeG2 cells were analyzed for (**A**) dihydroxy- and (**B**) epoxy-PUFA in the two lipid classes neutral lipids (NL) and phospholipids (PL). PL were separated from NL on an amino propyl silica phase based solid phase extraction followed by quantification of total oxylipins after alkaline hydrolysis by LC-MS/MS. Shown are concentrations [pmol/mg protein] of total oxylipins of (A) *erythro-* and *threo*-isomers of 5,6- and 11,12-DiHETrE, 4,5- and 10,11-DiHDPE as well as (B) *trans-* and *cis*-isomers of 11(12)-EpETrE and 10(11)-EpDPE for the classes of NL and PL as well as the sum of both lipid classes (NL + PL).

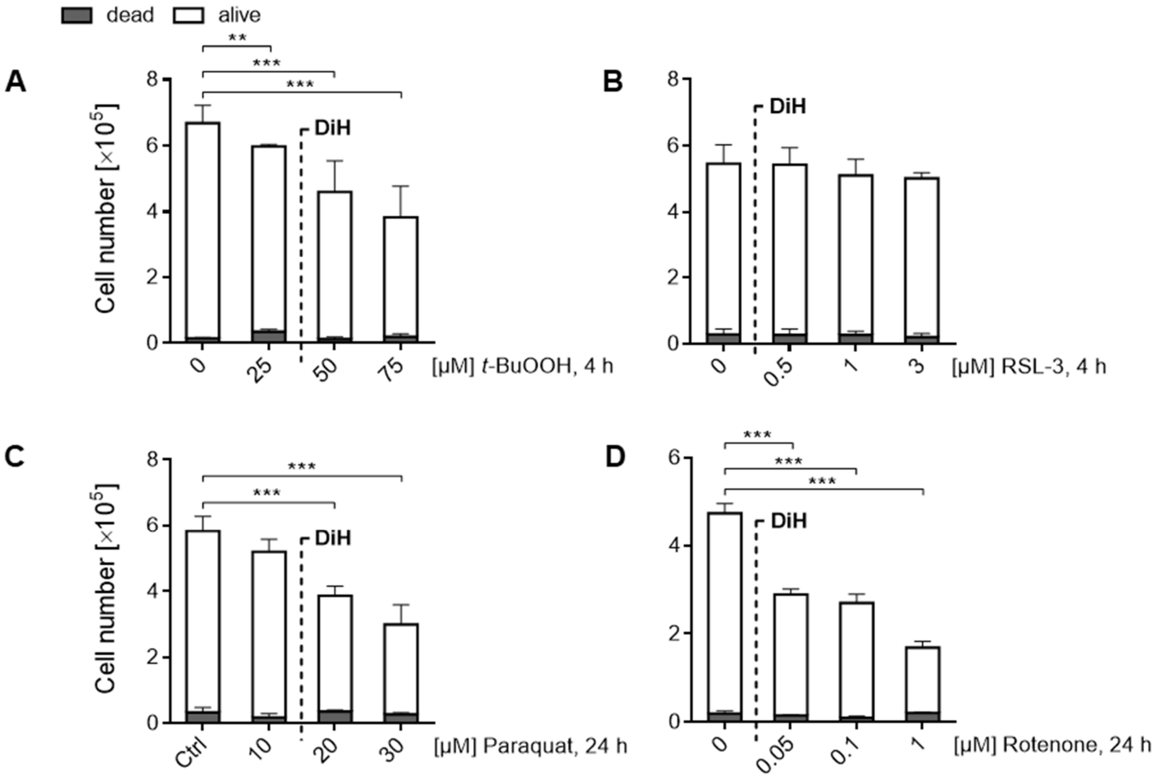

Fig. S7: Oxidative stress limits cell proliferation. Cell proliferation was used as biological endpoint to determine oxidative stress in different models of oxidative stress. Proliferation of HepG2 cells treated with (**A**) *t*-BuOOH, (**C**) paraquat and (**D**) rotenone was affected significantly at low doses. Proliferation of RSL-3 treated HepG2 cells was slightly but not significantly affected (**B**). All concentrations were below cytotoxic levels determined based on metabolic activity (resazurin), lysosomal integrity (neutral red) and membrane integrity (LDH leakage assay). Concentrations of the stimuli at which significant increases of dihydroxy-PUFA (see Fig. 4) are observed are indicated with **DiH** (dotted line). Absolute cell numbers of living (white bars) and dead (grey bars) cells were determined using a Neubauer chamber (mean ± SD, n = 3). Statistical significances were determined using one-way-ANOVA following Fisher's Least Significant Differences test (* $p < 0.05$, ** $p < 0.01$, *** $p < 0.001$).

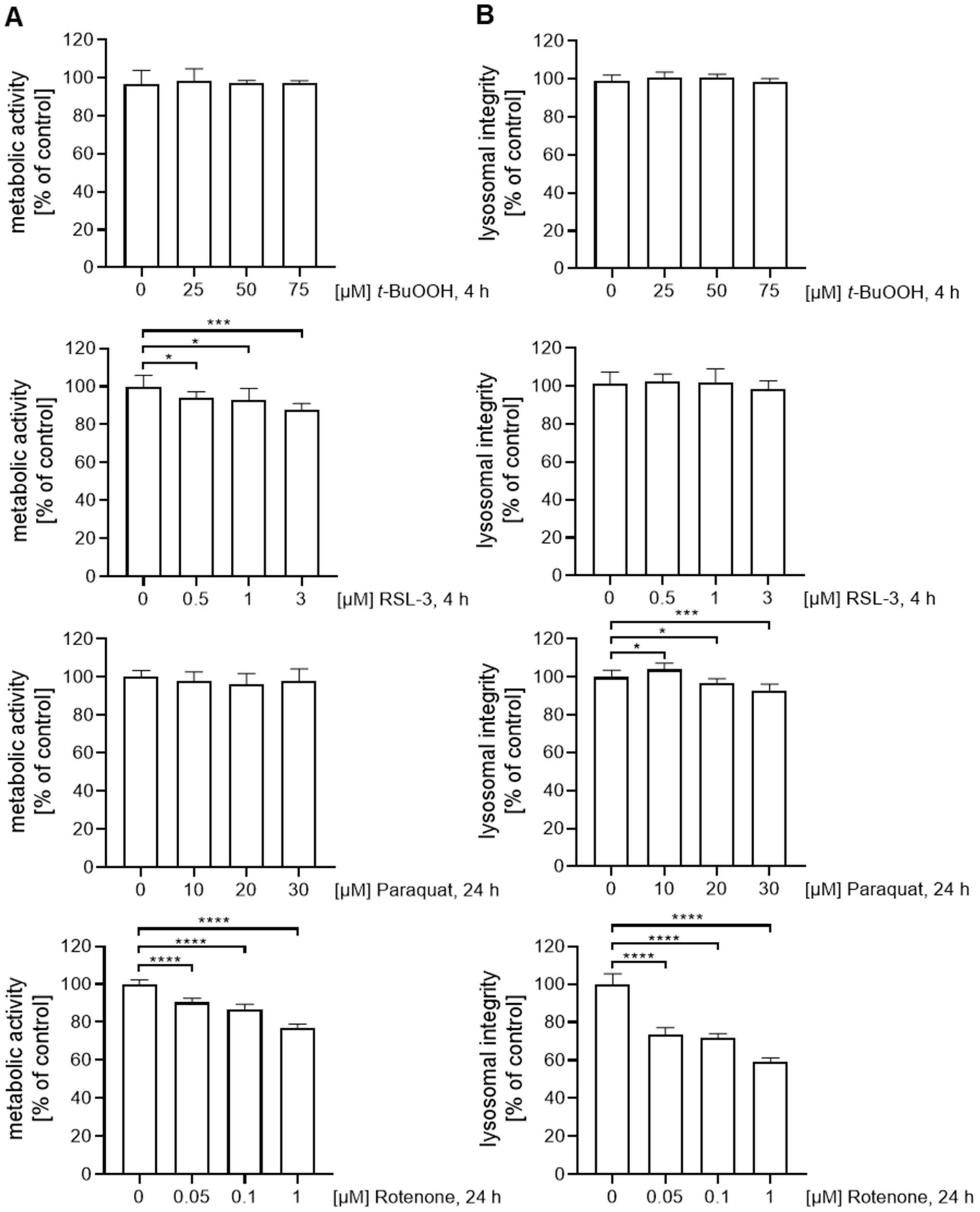

Fig. S8: Cell viability of HepG2 cells in the four models of oxidative stress tested by (**A**) resazurin (alamar blue) assay for metabolic activity and (**B**) neutral red uptake assay for lysosomal integrity. HepG2 cells were treated with t-BuOOH, RSL-3, paraquat and rotenone for the indicated concentrations and periods of time. Results are shown normalized to the vehicle control (mean ± SD, n = 6-12). Statistical significances were determined using one-way-ANOVA following Fisher's Least Significant Differences test (* p < 0.05, ** p < 0.01, *** p < 0.001, **** p < 0.0001).

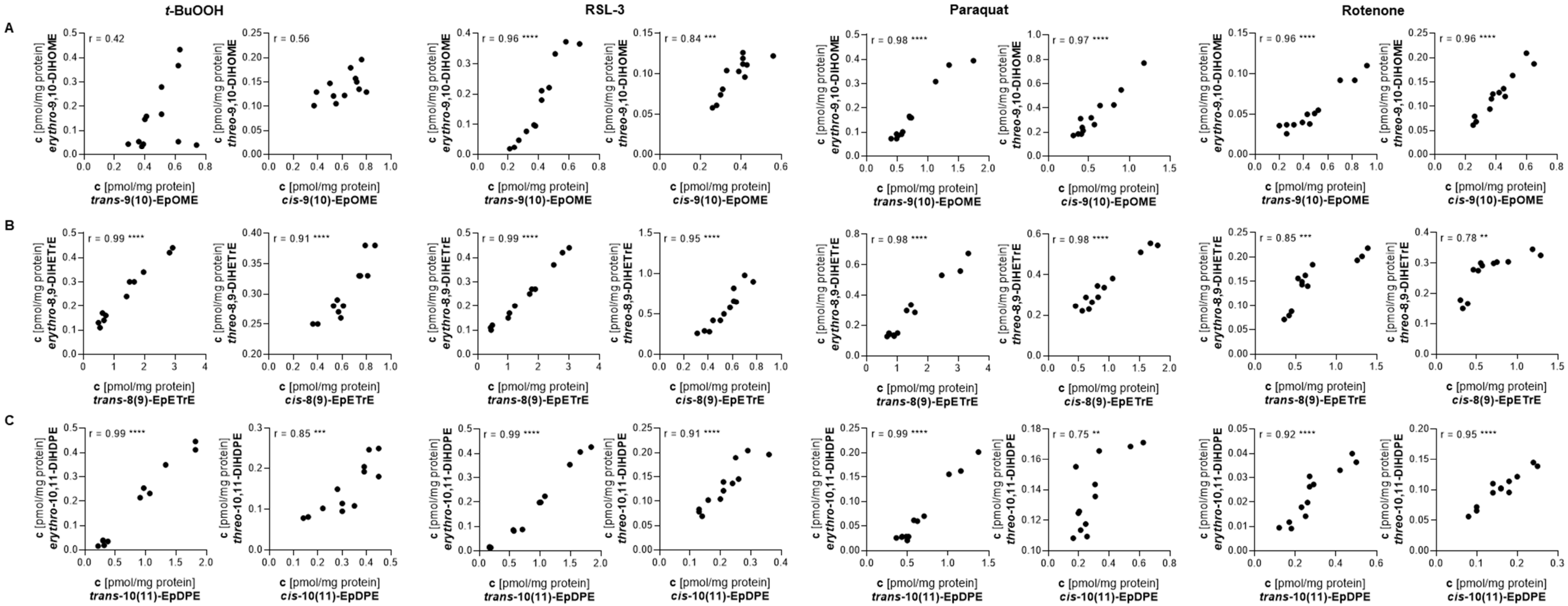

Fig. S9: Correlation of *erythro*-dihydroxy-PUFA to *trans*-epoxy-PUFA concentrations as well as *threo*-dihydroxy-PUFA to *cis*-epoxy-PUFA concentrations. Concentrations of *erythro*- and *threo*- (A) 9,10-DiHOME, (B) 8,9-DiHETrE and (C) 10,11-DiHDPE are compared with concentrations of *trans*- and *cis*- (A) 9(10)-EpOME, (B) 8(9)-EpETrE and (C) 10(11)-EpDPE in different models of oxidative stress in HepG2 cells: *tert*-butyl hydroperoxide (0–75 µM, 4 h, PBS as vehicle control), RSL-3 (0–3 µM, 4h, 0.1% DMSO as vehicle control), paraquat (0–30 µM, 24 h, PBS as vehicle control) and rotenone (0–1 µM, 24 h, 0.1% DMSO as vehicle control). Total oxylipin concentrations in the cells were determined by LC-MS/MS. Correlations were calculated using Pearson's r as indicated (** $p < 0.01$, *** $p < 0.001$, **** $p < 0.0001$).

4. Supplementary tables

Tab. S1: Parameters of the LC-ESI(-)-MS/MS method for the quantification of *erythro-* and *threo-*dihydroxy-PUFA, *cis-* and *trans-*epoxy-PUFA and selected isoprostanes. Shown are retention times, mass transitions for quantification in scheduled SRM mode, the assigned internal standards and external calibration parameters. References refer to *threo*-dihydroxy-PUFA or *cis*-epoxy-PUFA.

	precursor-PUFA	retention time [min]		mass transitions *m/z*		internal standard	calibration (weighting: $1/x^2$)					reference
				Q1	Q3		LLOQ [nM]	ULOQ* [nM]	r	slope	y-intercept	
**vicinal dihydroxy-PUFA**		***erythro-***	***threo-***									
9,10-DiHOME	LA	14.16	15.01	312.2	201.2	$^2H_4$-9,10-DiHOME	0.25	1000	0.999	0.177	0.0220	[31]
12,13-DiHOME	LA	13.62	14.55	313.2	183.2	$^2H_4$-9,10-DiHOME	0.25	1000	0.999	0.162	0.0124	[31]
5,6-DiHETrE	ARA	17.65	18.18	337.2	145.1	$^2H_{11}$-11,12-DiHETrE	0.25	750	0.998	0.0454	0.00438	[31]
8,9-DiHETrE	ARA	16.40	17.24	337.2	127.1	$^2H_{11}$-11,12-DiHETrE	0.50	1000	0.999	0.0359	0.00242	[31]
11,12-DiHETrE	ARA	15.71	16.58	337.2	167.1	$^2H_{11}$-11,12-DiHETrE	0.50	500	0.999	0.0773	0.00431	[31]
14,15-DiHETrE	ARA	14.89	15.77	337.2	207.1	$^2H_{11}$-11,12-DiHETrE	0.25	250	0.999	0.121	0.00179	[31]
4,5-DiHDPE	DHA	18.94	19.30	361.2	229.3	$^2H_{11}$-11,12-DiHETrE	*relative quantification based on 7,8-DiHDPE*					[25]
7,8-DiHDPE	DHA	17.28	17.97	361.2	113.1	$^2H_{11}$-11,12-DiHETrE	2.5	1000	1.000	0.0179	0.00262	[31]
10,11-DiHDPE	DHA	16.32	17.14	361.2	153.2	$^2H_{11}$-11,12-DiHETrE	0.25	500	1.000	0.0567	0.00422	[31]
13,14-DiHDPE	DHA	15.90	16.76	361.2	193.2	$^2H_{11}$-11,12-DiHETrE	0.50	500	1.000	0.0570	0.00245	[31]
16,17-DiHDPE	DHA	15.64	16.47	361.2	233.2	$^2H_{11}$-11,12-DiHETrE	0.50	1000	0.999	0.0475	0.00138	[31]
19,20-DiHDPE	DHA	15.05	15.83	361.2	273.2	$^2H_{11}$-11,12-DiHETrE	1.0	1000	1.000	0.0288	0.00334	[31]
**epoxy-PUFA**		***trans-***	***cis-***									
9(10)-EpOME	LA	22.83	22.59	295.2	171.1	$^2H_4$-12(13)-EpOME	1.0	1000	1.000	0.0799	0.00554	[26, 31]
12(13)-EpOME	LA	22.64	22.41	295.2	195.2	$^2H_4$-12(13)-EpOME	2.5	1000	1.000	0.0355	0.0270	[26, 31]
8(9)-EpETrE	ARA	23.49	23.23	319.2	155.2	$^2H_{11}$-8(9)-EpETrE	5.0	1000	1.000	0.0815	0.0619	[26, 31]
11(12)-EpETrE	ARA	23.32	23.07	319.2	167.2	$^2H_{11}$-8(9)-EpETrE	0.25	1000	0.998	0.222	0.0159	[26, 31]
14(15)-EpETrE	ARA	22.83	22.56	319.2	219.2	$^2H_{11}$-14(15)-EpETrE	0.75	1000	0.999	0.0610	0.0122	[26, 31]

Tab. S1: continued.

7(8)-EpDPE	DHA	23.38	23.15	343.2	141.2	$^{2}H_{11}$-8(9)-EpETrE	2.5	1000	0.999	0.0441	0.0462	[26, 31]
10(11)-EpDPE	DHA	23.23	22.97	343.2	153.2	$^{2}H_{11}$-8(9)-EpETrE	0.10	750	0.999	0.312	0.00203	[26, 31]
13(14)-EpDPE	DHA	23.12	22.87	343.2	193.2	$^{2}H_{11}$-14(15)-EpETrE	0.75	1000	0.998	0.0504	0.00816	[26, 31]
16(17)-EpDPE	DHA	22.99	22.79	343.2	233.2	$^{2}H_{11}$-14(15)-EpETrE	0.75	1000	0.998	0.0421	0.00538	[26, 31]
19(20)-EpDPE	DHA	22.51	22.27	343.2	241.2	$^{2}H_{11}$-14(15)-EpETrE	1.000	1000	1.000	0.0318	0.00657	[26, 31]
**isoprostanes**												
5-iPF$_{2\alpha}$-VI (5(*R*,*S*)-5-F$_{2t}$-IsoP)	ARA	8.04		353.2	114.8	$^{2}H_{4}$-8-iso-PGF$_{2\alpha}$	0.75	1000	0.997	0.129	0.0179	[31]
8-iso-PGF$_{2\alpha}$ (15-F$_{2t}$-IsoP)	ARA	7.45		353.1	193.1	$^{2}H_{4}$-8-iso-PGF$_{2\alpha}$	0.75	1000	0.998	0.129	0.0149	[31]
8,12-iso-iPF$_{2\alpha}$-VI (5(*R*,*S*)-5-F$_{2c}$-IsoP)	ARA	10.04		353.2	219.2	$^{2}H_{11}$-8,12-iso-iPF$_{2\alpha}$-VI	2.5	1000	0.999	0.0177	0.0135	[31]

* highest calibration level at 1000 nM

Tab. S2: Free oxylipins in models of oxidative stress in HepG2 cells. Shown are concentrations of non-esterified vicinal dihydroxy-PUFA, epoxy-PUFA and isoprostanes (pmol/mg protein, mean ± SD, n = 3) in all tested models of oxidative stress. Oxylipins were extracted and quantified after solid-phase extraction using targeted LC-MS/MS. The average protein amount was in a range of 300-800 µg protein per sample.

c [pmol/mg protein]	***t*-BuOOH**		**RSL-3**		**Paraquat**		**Rotenone**	
	control<br>50 µM, 4 h		control<br>1 µM, 4h		control<br>30 µM, 24 h		control<br>1 µM, 4 h	
**vicinal dihydroxy-PUFA**	***erythro-***	***threo-***	***erythro-***	***threo-***	***erythro-***	***threo-***	***erythro-***	***threo-***
9,10-DiHOME	<LLOQ<br>0.07 ± 0.07	0.032 ± 0.006<br>0.08 ± 0.08	<LLOQ<br>0.007 ± 0.003	0.019 ± 0.001<br>0.0170 ± 0.0003	<LLOQ<br><LLOQ	0.015 ± 0.001<br>0.020 ± 0.003	0.016 ± 0.005<br>0.020 ± 0.004	0.027 ± 0.003<br>0.026 ± 0.006
12,13-DiHOME	<LLOQ<br><LLOQ	0.015 ± 0.001<br>0.03 ± 0.03	<LLOQ<br><LLOQ	0.009 ± 0.002<br>0.010 ± 0.003	<LLOQ<br><LLOQ	0.006 ± 0.001<br>0.010 ± 0.002	<LLOQ<br><LLOQ	0.013 ± 0.001<br>0.012 ± 0.003
5,6-DiHETrE	<LLOQ<br><LLOQ	<LLOQ<br><LLOQ	<LLOQ<br><LLOQ	<LLOQ<br><LLOQ	<LLOQ<br><LLOQ	<LLOQ<br><LLOQ	<LLOQ<br><LLOQ	<LLOQ<br><LLOQ
8,9-DiHETrE	<LLOQ<br><LLOQ	<LLOQ<br><LLOQ	<LLOQ<br><LLOQ	<LLOQ<br><LLOQ	<LLOQ<br><LLOQ	<LLOQ<br><LLOQ	<LLOQ<br><LLOQ	<LLOQ<br><LLOQ
11,12-DiHETrE	<LLOQ<br><LLOQ	<LLOQ<br><LLOQ	<LLOQ<br><LLOQ	<LLOQ<br><LLOQ	<LLOQ<br><LLOQ	<LLOQ<br><LLOQ	<LLOQ<br><LLOQ	<LLOQ<br><LLOQ
14,15-DiHETrE	<LLOQ<br>0.006 ± 0.001	<LLOQ<br>0.006 ± 0.003	<LLOQ<br><LLOQ	<LLOQ<br><LLOQ	<LLOQ<br><LLOQ	<LLOQ<br><LLOQ	<LLOQ<br><LLOQ	<LLOQ<br><LLOQ
4,5-DiHDPE	<LLOQ<br><LLOQ	<LLOQ<br><LLOQ	<LLOQ<br><LLOQ	<LLOQ<br><LLOQ	<LLOQ<br><LLOQ	<LLOQ<br><LLOQ	<LLOQ<br><LLOQ	<LLOQ<br><LLOQ
7,8-DiHDPE	<LLOQ<br><LLOQ	<LLOQ<br><LLOQ	<LLOQ<br><LLOQ	<LLOQ<br><LLOQ	<LLOQ<br><LLOQ	<LLOQ<br><LLOQ	<LLOQ<br><LLOQ	<LLOQ<br><LLOQ
10,11-DiHDPE	<LLOQ<br><LLOQ	<LLOQ<br><LLOQ	<LLOQ<br><LLOQ	<LLOQ<br><LLOQ	<LLOQ<br><LLOQ	<LLOQ<br><LLOQ	<LLOQ<br><LLOQ	<LLOQ<br><LLOQ
13,14-DiHDPE	<LLOQ<br><LLOQ	<LLOQ<br><LLOQ	<LLOQ<br><LLOQ	<LLOQ<br><LLOQ	<LLOQ<br><LLOQ	<LLOQ<br><LLOQ	<LLOQ<br><LLOQ	<LLOQ<br><LLOQ
16,17-DiHDPE	<LLOQ<br><LLOQ	<LLOQ<br><LLOQ	<LLOQ<br><LLOQ	<LLOQ<br><LLOQ	<LLOQ<br><LLOQ	<LLOQ<br><LLOQ	<LLOQ<br><LLOQ	<LLOQ<br><LLOQ
19,20-DiHDPE	<LLOQ<br><LLOQ	<LLOQ<br><LLOQ	<LLOQ<br><LLOQ	<LLOQ<br><LLOQ	<LLOQ<br><LLOQ	<LLOQ<br><LLOQ	<LLOQ<br><LLOQ	<LLOQ<br><LLOQ

Tab. S2: continued.

**epoxy-PUFA**	***trans-***	***cis-***	***trans-***	***cis-***	***trans-***	***cis-***	***trans-***	***cis-***
9(10)-EpOME	0.12 ± 0.05<br>0.09 ± 0.04	0.09 ± 0.03<br>0.08 ± 0.06	0.06 ± 0.01<br>0.050 ± 0.009	0.04 ± 0.01<br>0.036 ± 0.004	0.052 ± 0.004<br>0.07 ± 0.01	0.04 ± 0.01<br>0.050 ± 0.003	0.070 ± 0.003<br>0.07 ± 0.01	0.05 ± 0.01<br>0.043 ± 0.009
12(13)-EpOME	0.08 ± 0.03<br>0.06 ± 0.04	0.08 ± 0.02<br>0.07 ± 0.05	0.04 ± 0.01<br>0.04 ± 0.01	0.02 ± 0.01<br>0.02 ± 0.01	0.029 ± 0.006<br>0.05 ± 0.01	0.03 ± 0.01<br>0.034 ± 0.005	0.061 ± 0.009<br>0.06 ± 0.01	0.020 ± 0.005<br>0.031 ± 0.004
8(9)-EpETrE	<LLOQ<br><LLOQ	<LLOQ<br><LLOQ	<LLOQ<br><LLOQ	<LLOQ<br><LLOQ	<LLOQ<br><LLOQ	<LLOQ<br><LLOQ	<LLOQ<br><LLOQ	<LLOQ<br><LLOQ
11(12)-EpETrE	<LLOQ<br><LLOQ	<LLOQ<br><LLOQ	<LLOQ<br><LLOQ	<LLOQ<br><LLOQ	<LLOQ<br><LLOQ	<LLOQ<br><LLOQ	<LLOQ<br><LLOQ	<LLOQ<br><LLOQ
14(15)-EpETrE	<LLOQ<br><LLOQ	<LLOQ<br><LLOQ	<LLOQ<br><LLOQ	<LLOQ<br><LLOQ	<LLOQ<br><LLOQ	<LLOQ<br><LLOQ	<LLOQ<br><LLOQ	<LLOQ<br><LLOQ
7(8)-EpDPE	<LLOQ<br><LLOQ	<LLOQ<br><LLOQ	<LLOQ<br><LLOQ	<LLOQ<br><LLOQ	<LLOQ<br><LLOQ	<LLOQ<br><LLOQ	<LLOQ<br><LLOQ	<LLOQ<br><LLOQ
10(11)-EpDPE	<LLOQ<br><LLOQ	<LLOQ<br><LLOQ	<LLOQ<br><LLOQ	<LLOQ<br><LLOQ	<LLOQ<br><LLOQ	<LLOQ<br><LLOQ	<LLOQ<br><LLOQ	<LLOQ<br><LLOQ
13(14)-EpDPE	<LLOQ<br><LLOQ	<LLOQ<br><LLOQ	<LLOQ<br><LLOQ	<LLOQ<br><LLOQ	<LLOQ<br><LLOQ	<LLOQ<br><LLOQ	<LLOQ<br><LLOQ	<LLOQ<br><LLOQ
16(17)-EpDPE	<LLOQ<br><LLOQ	<LLOQ<br><LLOQ	<LLOQ<br><LLOQ	<LLOQ<br><LLOQ	<LLOQ<br><LLOQ	<LLOQ<br><LLOQ	<LLOQ<br><LLOQ	<LLOQ<br><LLOQ
19(20)-EpDPE	<LLOQ<br><LLOQ	<LLOQ<br><LLOQ	<LLOQ<br><LLOQ	<LLOQ<br><LLOQ	<LLOQ<br><LLOQ	<LLOQ<br><LLOQ	<LLOQ<br><LLOQ	<LLOQ<br><LLOQ
**isoprostanes**								
5-iPF$_{2\alpha}$-VI<br>(5(*R*,*S*)-5-F$_{2t}$-IsoP)	<LLOQ<br><LLOQ		<LLOQ<br><LLOQ		<LLOQ<br><LLOQ		<LLOQ<br><LLOQ	
8-iso-PGF$_{2\alpha}$<br>(15-F$_{2t}$-IsoP)	<LLOQ<br><LLOQ		<LLOQ<br><LLOQ		<LLOQ<br><LLOQ		<LLOQ<br><LLOQ	
8,12-iso-iPF$_{2\alpha}$-VI<br>(5(*R*,*S*)-5-F$_{2c}$-IsoP)	<LLOQ<br><LLOQ		<LLOQ<br><LLOQ		<LLOQ<br><LLOQ		<LLOQ<br><LLOQ	

Tab. S3: Total oxylipins in models of oxidative stress. Shown are concentrations of total vicinal dihydroxy-PUFA, epoxy-PUFA and isoprostanes (pmol/mg protein, mean ± SD, n = 3) in all tested models of oxidative stress. Following extraction and alkaline hydrolysis, total oxylipins were quantified after solid phase extraction using targeted LC-MS/MS. Average protein amount was in a range of 300-800 µg protein per sample. Since concentrations of free oxylipins are low (Table S2), concentrations of total oxylipins represent concentration of esterified oxylipins.

c [pmol/mg protein]	***t*-BuOOH**		**RSL-3**		**Paraquat**		**Rotenone**	
	control<br>25 µM, 4 h<br>50 µM, 4 h<br>75 µM, 4 h		control<br>500 nM, 4 h<br>1 µM, 4 h<br>3 µM, 4h		control<br>10 µM, 24 h<br>20 µM, 24 h<br>30 µM, 24 h		control<br>50 nM, 24 h<br>100 nM, 24 h<br>1 µM, 24 h	
**vicinal dihydroxy-PUFA**	***erythro-***	***threo-***	***erythro-***	***threo-***	***erythro-***	***threo-***	***erythro-***	***threo-***
9,10-DiHOME	0.039 ± 0.005	0.12 ± 0.01	0.03 ± 0.02	0.07 ± 0.01	0.092 ± 0.008	0.24 ± 0.06	0.033 ± 0.006	0.069 ± 0.009
	0.050 ± 0.006	0.12 ± 0.02	0.09 ± 0.01	0.09 ± 0.02	0.079 ± 0.011	0.19 ± 0.02	0.047 ± 0.009	0.12 ± 0.02
	0.16 ± 0.01	0.14 ± 0.01	0.20 ± 0.02	0.107 ± 0.005	0.162 ± 0.003	0.33 ± 0.08	0.042 ± 0.007	0.120 ± 0.005
	0.36 ± 0.08	0.17 ± 0.02	0.36 ± 0.02	0.123 ± 0.003	0.36 ± 0.05	0.6 ± 0.2	0.10 ± 0.01	0.19 ± 0.02
12,13-DiHOME	0.017 ± 0.003	0.065 ± 0.003	0.007 ± 0.002	0.036 ± 0.006	0.038 ± 0.002	0.16 ± 0.02	0.0095 ± 0.0009	0.040 ± 0.005
	0.017 ± 0.004	0.07 ± 0.01	0.014 ± 0.0008	0.05 ± 0.01	0.038 ± 0.01	0.13 ± 0.02	0.017 ± 0.005	0.07 ± 0.01
	0.0186 ± 0.0009	0.064 ± 0.008	0.015 ± 0.002	0.0388 ± 0.0006	0.065 ± 0.009	0.22 ± 0.03	0.013 ± 0.002	0.06 ± 0.01
	0.026 ± 0.004	0.063 ± 0.006	0.205 ± 0.0004	0.039 ± 0.003	0.119 ± 0.037	0.4 ± 0.1	0.026 ± 0.005	0.10 ± 0.01
5,6-DiHETrE	0.18 ± 0.03	0.34 ± 0.06	0.15 ± 0.01	0.28 ± 0.01	0.23 ± 0.03	0.43 ± 0.03	0.14 ± 0.03	0.29 ± 0.04
	0.25 ± 0.02	0.39 ± 0.05	0.530 ± 0.05	0.45 ± 0.05	0.30 ± 0.03	0.48 ± 0.03	0.25 ± 0.02	0.54 ± 0.02
	0.8 ± 0.07	0.64 ± 0.07	0.92 ± 0.04	0.63 ± 0.04	0.6 ± 0.1	0.9 ± 0.1	0.29 ± 0.02	0.60 ± 0.02
	1.4 ± 0.2	0.9 ± 0.1	1.5 ± 0.1	0.90 ± 0.08	1.4 ± 0.1	1.6 ± 0.2	0.543 ± 0.008	0.80 ± 0.05
8,9-DiHETrE	0.13 ± 0.01	0.26 ± 0.02	0.108 ± 0.008	0.2 ± 0.01	0.130 ± 0.004	0.25 ± 0.02	0.079 ± 0.008	0.16 ± 0.01
	0.16 ± 0.01	0.28 ± 0.01	0.180 ± 0.02	0.22 ± 0.02	0.146 ± 0.004	0.27 ± 0.04	0.144 ± 0.004	0.282 ± 0.009
	0.28 ± 0.03	0.31 ± 0.03	0.27 ± 0.01	0.247 ± 0.008	0.31 ± 0.03	0.35 ± 0.02	0.17 ± 0.01	0.301 ± 0.002
	0.40 ± 0.05	0.36 ± 0.03	0.41 ± 0.04	0.30 ± 0.03	0.59 ± 0.08	0.54 ± 0.02	0.20 ± 0.01	0.32 ± 0.02

Tab. S3: continued.

11,12-DiHETrE	0.020 ± 0.003	0.07 ± 0.01	0.018 ± 0.001	0.045 ± 0.001	0.037 ± 0.009	0.157 ± 0.008	0.016 ± 0.001	0.027 ± 0.004
	0.035 ± 0.002	0.087 ± 0.009	0.105 ± 0.009	0.076 ± 0.009	0.042 ± 0.007	0.16 ± 0.01	0.031 ± 0.001	0.069 ± 0.006
	0.18 ± 0.02	0.13 ± 0.01	0.21 ± 0.01	0.093 ± 0.005	0.079 ± 0.006	0.128 ± 0.007	0.033 ± 0.002	0.061 ± 0.008
	0.37 ± 0.07	0.17 ± 0.03	0.36 ± 0.03	0.118 ± 0.008	0.20 ± 0.02	0.18 ± 0.03	0.062 ± 0.005	0.07 ± 0.01
14,15-DiHETrE	<LLOQ	0.016 ± 0.002	<LLOQ	<LLOQ	<LLOQ	0.051 ± 0.008	<LLOQ	<LLOQ
	0.010 ± 0.002	0.019 ± 0.003	0.026 ± 0.003	0.021 ± 0.001	<LLOQ	0.053 ± 0.007	<LLOQ	<LLOQ
	0.042 ± 0.002	0.034 ± 0.004	0.047 ± 0.004	0.032 ± 0.003	<LLOQ	0.055 ± 0.003	<LLOQ	<LLOQ
	0.09 ± 0.02	0.06 ± 0.01	0.077 ± 0.006	0.041 ± 0.006	0.099 ± 0.006	0.061 ± 0.007	<LLOQ	<LLOQ
4,5-DiHDPE	0.22 ± 0.05	0.22 ± 0.04	0.21 ± 0.02	0.187 ± 0.009	0.41 ± 0.01	0.318 ± 0.007	0.18 ± 0.05	0.18 ± 0.02
	0.530 ± 0.08	0.30 ± 0.03	1.400 ± 0.1	0.41 ± 0.05	0.58 ± 0.08	0.34 ± 0.05	0.33 ± 0.04	0.24 ± 0.01
	2.9 ± 0.3	0.78 ± 0.08	2.8 ± 0.1	0.71 ± 0.02	0.9 ± 0.1	0.40 ± 0.03	0.38 ± 0.02	0.254 ± 0.003
	4.9 ± 0.6	1.2 ± 0.2	4.7 ± 0.3	1.09 ± 0.07	1.8 ± 0.1	0.64 ± 0.03	0.76 ± 0.03	0.387 ± 0.008
7,8-DiHDPE	0.10 ± 0.01	0.087 ± 0.008	0.09 ± 0.01	0.08 ± 0.01	<LLOQ	<LLOQ	<LLOQ	<LLOQ
	0.150 ± 0.04	0.11 ± 0.01	0.190 ± 0.03	0.11 ± 0.01	<LLOQ	<LLOQ	<LLOQ	<LLOQ
	0.4 ± 0.05	0.18 ± 0.03	0.36 ± 0.02	0.16 ± 0.02	<LLOQ	<LLOQ	<LLOQ	<LLOQ
	0.59 ± 0.04	0.26 ± 0.04	0.62 ± 0.09	0.26 ± 0.03	<LLOQ	<LLOQ	<LLOQ	<LLOQ
10,11-DiHDPE	0.018 ± 0.002	0.09 ± 0.01	0.014 ± 0.002	0.077 ± 0.008	0.025 ± 0.004	0.14 ± 0.02	0.010 ± 0.001	0.064 ± 0.008
	0.037 ± 0.002	0.11 ± 0.01	0.084 ± 0.003	0.11 ± 0.01	0.027 ± 0.001	0.113 ± 0.005	0.017 ± 0.003	0.097 ± 0.004
	0.23 ± 0.02	0.17 ± 0.02	0.21 ± 0.01	0.14 ± 0.004	0.064 ± 0.006	0.13 ± 0.02	0.028 ± 0.002	0.109 ± 0.006
	0.40 ± 0.05	0.23 ± 0.03	0.39 ± 0.04	0.196 ± 0.008	0.17 ± 0.03	0.168 ± 0.003	0.036 ± 0.003	0.13 ± 0.01
13,14-DiHDPE	<LLOQ	0.028 ± 0.004	<LLOQ	<LLOQ	<LLOQ	0.09 ± 0.01	<LLOQ	<LLOQ
	0.018 ± 0.005	0.04 ± 0.005	0.074 ± 0.004	0.047 ± 0.004	<LLOQ	0.096 ± 0.008	<LLOQ	<LLOQ
	0.15 ± 0.02	0.07 ± 0.007	0.151 ± 0.005	0.063 ± 0.002	<LLOQ	0.08 ± 0.01	<LLOQ	<LLOQ
	0.31 ± 0.04	0.12 ± 0.02	0.28 ± 0.02	0.092 ± 0.005	<LLOQ	0.15 ± 0.03	<LLOQ	<LLOQ
16,17-DiHDPE	<LLOQ	<LLOQ	<LLOQ	<LLOQ	<LLOQ	<LLOQ	<LLOQ	<LLOQ
	<LLOQ	0.023 ± 0.004	0.050 ± 0.01	0.029 ± 0.008	<LLOQ	<LLOQ	<LLOQ	<LLOQ
	0.107 ± 0.006	0.050 ± 0.006	0.108 ± 0.002	0.033 ± 0.003	<LLOQ	<LLOQ	<LLOQ	<LLOQ
	0.22 ± 0.04	0.09 ± 0.02	0.19 ± 0.02	0.054 ± 0.003	<LLOQ	<LLOQ	<LLOQ	<LLOQ
19,20-DiHDPE	<LLOQ	<LLOQ	<LLOQ	<LLOQ	<LLOQ	<LLOQ	<LLOQ	<LLOQ
	<LLOQ	0.033 ± 0.006	<LLOQ	<LLOQ	<LLOQ	<LLOQ	<LLOQ	<LLOQ
	0.10 ± 0.02	0.052 ± 0.007	0.10 ± 0.02	<LLOQ	<LLOQ	<LLOQ	<LLOQ	<LLOQ
	0.21 ± 0.05	0.08 ± 0.01	0.19 ± 0.02	0.066 ± 0.002	<LLOQ	<LLOQ	<LLOQ	<LLOQ

Tab. S3: continued.

epoxy-PUFA	*trans-*	*cis-*	*trans-*	*cis-*	*trans-*	*cis-*	*trans-*	*cis-*
9(10)-EpOME	0.5 ± 0.2	0.43 ± 0.09	0.24 ± 0.03	0.28 ± 0.02	0.56 ± 0.04	0.40 ± 0.03	0.24 ± 0.033	0.259 ± 0.008
	0.4 ± 0.2	0.6 ± 0.1	0.36 ± 0.03	0.38 ± 0.07	0.46 ± 0.05	0.38 ± 0.06	0.441 ± 0.105	0.41 ± 0.049
	0.44 ± 0.06	0.7 ± 0.2	0.44 ± 0.03	0.38 ± 0.04	0.71 ± 0.02	0.58 ± 0.05	0.426 ± 0.032	0.402 ± 0.047
	0.59 ± 0.06	0.72 ± 0.04	0.59 ± 0.08	0.46 ± 0.09	1.4 ± 0.3	1.0 ± 0.2	0.813 ± 0.109	0.585 ± 0.072
12(13)-EpOME	0.5 ± 0.2	0.33 ± 0.08	0.19 ± 0.04	0.18 ± 0.02	0.49 ± 0.04	0.23 ± 0.04	0.22 ± 0.06	0.152 ± 0.004
	0.4 ± 0.2	0.47 ± 0.09	0.30 ± 0.03	0.26 ± 0.06	0.45 ± 0.01	0.22 ± 0.02	0.38 ± 0.08	0.29 ± 0.03
	0.38 ± 0.07	0.5 ± 0.1	0.35 ± 0.02	0.25 ± 0.02	0.9 ± 0.1	0.30 ± 0.04	0.34 ± 0.04	0.25 ± 0.03
	0.5 ± 0.07	0.53 ± 0.03	0.45 ± 0.05	0.30 ± 0.05	1.3 ± 0.2	0.49 ± 0.03	0.62 ± 0.07	0.41 ± 0.04
8(9)-EpETrE	0.56 ± 0.09	0.43 ± 0.09	0.44 ± 0.03	0.37 ± 0.05	0.8 ± 0.1	0.6 ± 0.1	0.41 ± 0.05	0.34 ± 0.05
	0.67 ± 0.06	0.59 ± 0.03	1.1 ± 0.1	0.49 ± 0.04	0.8 ± 0.2	0.7 ± 0.1	0.60 ± 0.04	0.52 ± 0.06
	1.5 ± 0.1	0.7 ± 0.1	1.79 ± 0.09	0.60 ± 0.02	1.4 ± 0.1	0.9 ± 0.1	0.62 ± 0.09	0.7 ± 0.1
	2.6 ± 0.5	0.80 ± 0.07	2.8 ± 0.3	0.69 ± 0.08	3.0 ± 0.4	1.7 ± 0.1	1.32 ± 0.07	1.1 ± 0.2
11(12)-EpETrE	0.5 ± 0.1	0.46 ± 0.07	0.30 ± 0.01	0.28 ± 0.01	0.8 ± 0.1	0.47 ± 0.04	0.28 ± 0.05	0.18 ± 0.02
	0.5 ± 0.1	0.65 ± 0.02	0.70 ± 0.09	0.37 ± 0.04	0.7 ± 0.1	0.43 ± 0.08	0.40 ± 0.04	0.31 ± 0.02
	0.99 ± 0.09	0.7 ± 0.1	1.04 ± 0.02	0.43 ± 0.05	1.06 ± 0.09	0.55 ± 0.04	0.46 ± 0.03	0.31 ± 0.03
	0.16 ± 0.3	0.78 ± 0.07	1.6 ± 0.1	0.50 ± 0.07	2.2 ± 0.5	0.8 ± 0.2	0.82 ± 0.05	0.46 ± 0.05
14(15)-EpETrE	0.34 ± 0.08	0.6 ± 0.1	0.19 ± 0.04	0.37 ± 0.02	0.50 ± 0.04	0.63 ± 0.04	0.18 ± 0.03	0.23 ± 0.02
	0.35 ± 0.05	0.88 ± 0.06	0.46 ± 0.07	0.46 ± 0.06	0.45 ± 0.06	0.59 ± 0.09	0.27 ± 0.02	0.40 ± 0.03
	0.67 ± 0.09	1 ± 0.3	0.67 ± 0.03	0.52 ± 0.08	0.6 ± 0.1	0.66 ± 0.03	0.30 ± 0.05	0.41 ± 0.09
	1.1 ± 0.2	1.03 ± 0.05	1.02 ± 0.05	0.59 ± 0.09	1.2 ± 0.2	0.9 ± 0.2	0.54 ± 0.06	0.57 ± 0.04
7(8)-EpDPE	0.26 ± 0.07	0.17 ± 0.04	0.17 ± 0.02	0.13 ± 0.01	0.59 ± 0.05	0.31 ± 0.05	0.14 ± 0.01	0.12 ± 0.02
	0.37 ± 0.04	0.32 ± 0.02	0.8 ± 0.1	0.24 ± 0.04	0.6 ± 0.1	0.37 ± 0.05	0.23 ± 0.03	0.18 ± 0.02
	1.4 ± 0.1	0.4 ± 0.1	1.52 ± 0.05	0.31 ± 0.03	0.9 ± 0.1	0.55 ± 0.09	0.24 ± 0.02	0.22 ± 0.03
	2.5 ± 0.4	0.57 ± 0.07	2.5 ± 0.2	0.44 ± 0.04	1.8 ± 0.3	0.9 ± 0.3	0.44 ± 0.07	0.24 ± 0.05
10(11)-EpDPE	0.25 ± 0.06	0.17 ± 0.04	0.17 ± 0.01	0.135 ± 0.005	0.47 ± 0.04	0.19 ± 0.01	0.15 ± 0.03	0.09 ± 0.01
	0.33 ± 0.04	0.32 ± 0.03	0.61 ± 0.08	0.19 ± 0.03	0.44 ± 0.08	0.21 ± 0.04	0.25 ± 0.02	0.16 ± 0.02
	0.98 ± 0.8	0.37 ± 0.9	1.02 ± 0.05	0.24 ± 0.03	0.64 ± 0.06	0.29 ± 0.03	0.28 ± 0.01	0.16 ± 0.02
	1.7 ± 0.3	0.42 ± 0.3	1.7 ± 0.2	0.30 ± 0.06	1.2 ± 0.2	0.5 ± 0.1	0.47 ± 0.04	0.23 ± 0.03
13(14)-EpDPE	0.2 ± 0.04	0.19 ± 0.03	0.11 ± 0.01	0.115 ± 0.004	0.27 ± 0.01	0.18 ± 0.02	0.10 ± 0.01	0.07 ± 0.02
	0.21 ± 0.03	0.3 ± 0.01	0.34 ± 0.05	0.16 ± 0.02	0.24 ± 0.03	0.16 ± 0.03	0.15 ± 0.02	0.12 ± 0.02
	0.52 ± 0.07	0.3 ± 0.1	0.51 ± 0.03	0.18 ± 0.01	0.36 ± 0.06	0.23 ± 0.01	0.17 ± 0.02	0.13 ± 0.01
	0.8 ± 0.1	0.35 ± 0.03	0.80 ± 0.07	0.20 ± 0.04	0.6 ± 0.1	0.4 ± 0.1	0.28 ± 0.05	0.19 ± 0.04

Tab. S3: continued.

16(17)-EpDPE	0.16 ± 0.04<br>0.18 ± 0.02<br>0.41 ± 0.05<br>0.69 ± 0.08	0.21 ± 0.02<br>0.33 ± 0.02<br>0.3 ± 0.1<br>0.35 ± 0.03	0.11 ± 0.03<br>0.27 ± 0.04<br>0.42 ± 0.02<br>0.64 ± 0.06	0.134 ± 0.007<br>0.16 ± 0.04<br>0.17 ± 0.01<br>0.18 ± 0.04	0.27 ± 0.02<br>0.220 ± 0.002<br>0.29 ± 0.02<br>0.57 ± 0.08	0.21 ± 0.02<br>0.16 ± 0.03<br>0.175 ± 0.008<br>0.21 ± 0.02	0.09 ± 0.02<br>0.14 ± 0.03<br>0.148 ± 0.009<br>0.26 ± 0.04	0.09 ± 0.01<br>0.145 ± 0.001<br>0.14 ± 0.03<br>0.20 ± 0.05
19(20)-EpDPE	0.26 ± 0.06<br>0.28 ± 0.03<br>0.61 ± 0.07<br>0.9 ± 0.1	0.27 ± 0.08<br>0.67 ± 0.03<br>2 ± 0.3<br>2.20 ± 0.1	0.17 ± 0.02<br>0.38 ± 0.03<br>0.53 ± 0.03<br>0.75 ± 0.07	0.32 ± 0.04<br>0.48 ± 0.05<br>0.50 ± 0.02<br>0.49 ± 0.08	0.49 ± 0.05<br>0.40 ± 0.07<br>0.7 ± 0.1<br>1.1 ± 0.3	0.48 ± 0.07<br>0.56 ± 0.07<br>0.8 ± 0.1<br>1.6 ± 0.2	0.15 ± 0.02<br>0.23 ± 0.02<br>0.31 ± 0.02<br>0.43 ± 0.05	0.30 ± 0.07<br>0.47 ± 0.02<br>0.53 ± 0.04<br>0.8 ± 0.1
**isoprostanes**								
5-iPF$_{2\alpha}$-VI<br>(5(*R*,*S*)-5-F$_{2t}$-IsoP)	0.18 ± 0.05<br>0.28 ± 0.04<br>0.72 ± 0.08<br>1.2 ± 0.2		0.163 ± 0.003<br>0.33 ± 0.06<br>0.46 ± 0.01<br>0.56 ± 0.05		0.22 ± 0.01<br>0.29 ± 0.04<br>0.45 ± 0.06<br>0.7 ± 0.1		0.17 ± 0.02<br>0.24 ± 0.04<br>0.27 ± 0.02<br>0.43 ± 0.01	
8-iso-PGF$_{2\alpha}$<br>(15-F$_{2t}$-IsoP)	0.04 ± 0.02<br>0.06 ± 0.01<br>0.13 ± 0.02<br>0.23 ± 0.06		0.024 ± 0.007<br>0.0529 ± 0.0005<br>0.070 ± 0.002<br>0.102 ± 0.007		<LLOQ<br><LLOQ<br><LLOQ<br><LLOQ		<LLOQ<br><LLOQ<br><LLOQ<br><LLOQ	
8,12-iso-iPF$_{2\alpha}$-VI<br>(5(*R*,*S*)-5-F$_{2c}$-IsoP)	0.189 ± 0.006<br>0.34 ± 0.07<br>0.6 ± 0.1<br>1.0 ± 0.2		0.25 ± 0.05<br>0.37 ± 0.07<br>0.46 ± 0.04<br>0.61 ± 0.08		0.5 ± 0.1<br>0.48 ± 0.07<br>0.8 ± 0.1<br>1.7 ± 0.5		0.22 ± 0.01<br>0.15 ± 0.06<br>0.23 ± 0.05<br>0.48 ± 0.07	
